# Arrayed Cobalt Phosphide Electrocatalyst Achieves Low Energy Consumption and Persistent H_2_ Liberation from Anodic Chemical Conversion

**DOI:** 10.1007/s40820-020-00486-2

**Published:** 2020-07-22

**Authors:** Kai Zhang, Gong Zhang, Qinghua Ji, Jiuhui Qu, Huijuan Liu

**Affiliations:** 1 State Key Laboratory of Environmental Aquatic Chemistry, Research Center for Eco-Environmental Sciences, Chinese Academy of Sciences, Beijing, 100085 People’s Republic of China; 2 Center for Water and Ecology, State Key Joint Laboratory of Environment Simulation and Pollution Control, School of Environment, Tsinghua University, Beijing, 100084 People’s Republic of China; 3 Key Laboratory of Drinking Water Science and Technology, Research Center for Eco-Environmental Sciences, Chinese Academy of Sciences, Beijing, 100085 People’s Republic of China; 4 University of Chinese Academy of Sciences, Beijing, 100049 People’s Republic of China

**Keywords:** Electrocatalysis, Cobalt phosphide, Hydrogen purification, Ammonia oxidation reaction, Membrane-free architecture

## Abstract

Electrochemical reduction of water to hydrogen (H_2_) offers a promising strategy for production of clean energy, but the design and optimization of electrochemical apparatus present challenges in terms of H_2_ recovery and energy consumption. Using cobalt phosphide nanoarrays (Co_2_P/CoP NAs) as a charge mediator, we effectively separated the H_2_ and O_2_ evolution of alkaline water electrolysis in time, thereby achieving a membrane-free pathway for H_2_ purification. The hierarchical array structure and synergistic optimization of the electronic configuration of metallic Co_2_P and metalloid CoP make the Co_2_P/CoP NAs high-efficiency bifunctional electrocatalysts for both charge storage and hydrogen evolution. Theoretical investigations revealed that the introduction of Co_2_P into CoP leads to a moderate hydrogen adsorption free energy and low water dissociation barrier, which are beneficial for boosting HER activity. Meanwhile, Co_2_P/CoP NAs with high capacitance could maintain a cathodic H_2_ evolution time of 1500 s at 10 mA cm^−2^ driven by a low average voltage of 1.38 V. Alternatively, the energy stored in the mediator could be exhausted via coupling with the anodic oxidation of ammonia, whereby only 0.21 V was required to hold the current for 1188 s. This membrane-free architecture demonstrates the potential for developing hydrogen purification technology at low cost.

**Electronic supplementary material:**

The online version of this article (10.1007/s40820-020-00486-2) contains supplementary material, which is available to authorized users.

## Highlights

- A template approach for the synthesis of porous cobalt phosphide nanoarrays (Co_2_P/CoP NAs) is reported, which exhibits superior electrocatalytic activity and stability toward charge storage and hydrogen evolution.
- Using Co_2_P/CoP NAs as a charge mediator, the H_2_ and O_2_ evolution of alkaline water electrolysis is separated effectively in time, thereby achieving a membrane-free pathway for H_2_ purification.
- Introduction of easily oxidized chemicals to replace water oxidation triggers a low energy consumption path toward H_2_ purification.

**Electronic supplementary material:**

The online version of this article (10.1007/s40820-020-00486-2) contains supplementary material, which is available to authorized users.

## Graphical abstract

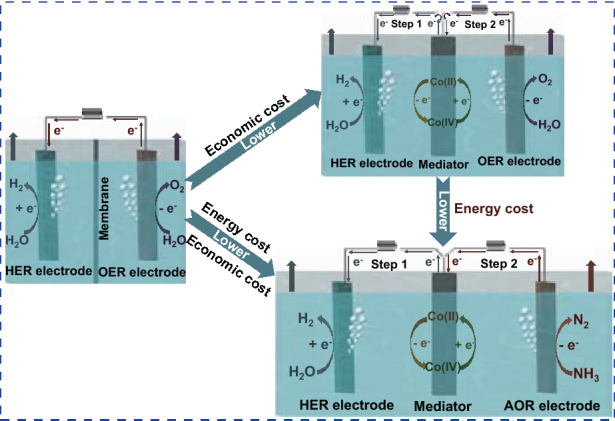

## Introduction

**Fig. 1:**
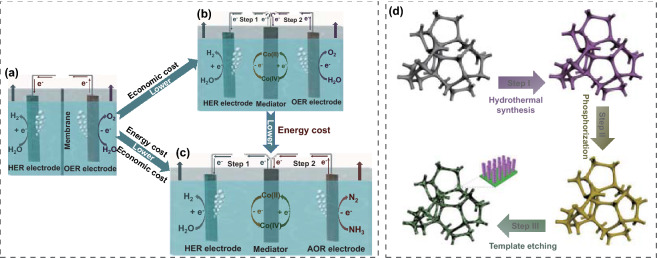
H_2_ production from different electrolytic cell architectures. **a** Conventional configuration of an alkaline water electrolysis cell with membrane. **b** Membrane-free configuration for stepwise HER and OER in alkaline electrolyte. **c** Membrane-free configuration for stepwise HER and ammonia oxidation reaction (AOR) in ammonia alkaline solutions. **d** Schematic illustration of the fabrication procedure of Co_2_P/CoP NAs electrocatalyst on CF

Electrochemical water splitting to produce hydrogen (H_2_) has been considered to be a sustainable and environmentally friendly energy conversion technology [1–3]. Currently, state-of-the-art water electrolyzers are based on proton-exchange membranes (PEMs) that separate H_2_ production and O_2_ production, but large-scale practical application remains restricted by their high cost and insufficient durability (Fig. 1a) [4–6]. Tremendous efforts have been focused on improvement in water electrolyzer architecture [7–10]. Unfortunately, design of a cost-effective electrochemical energy recovery apparatus to harvest a high-purity H_2_ stream for wide application remains a great challenge. Thus, innovative breakthroughs to enable water splitting cells with economical and stable H_2_ recovery are urgently needed.

Recently, using nickel hydroxide as a solid-state redox mediator, Xia and his coworkers achieved an alternative method to split conventional water electrolysis into two independent steps [11]. The production of O_2_ and H_2_ at separate points in time potentially reduces cost by decreasing the stringent demands placed on PEM-based water electrolysis (Fig. 1b). The finding paves the way toward an important pathway for separating H_2_ and O_2_ production in water electrolysis. Nevertheless, for the following two reasons, there is still great room for improvement in terms of the performance of the system. Firstly, the anodic reaction for oxygen evolution reaction (OER) in the counterpart cell requires a large overpotential on account of the intrinsically formidable kinetics, resulting in relatively low overall efficiency [12, 13]. Previous works have reported that more easily oxidized chemicals could be used as sacrificial agents for electrochemical oxidation to replace the OER, complementing the advancement of overall energy conversion efficiency during the electrolysis process [14–17]. Among these energy-saving electrolysis techniques, ammonia (NH_3_) electrolysis (2NH_3_ → 3H_2_ + N_2_) is attracting extensive attention due to the capability of simultaneous H_2_ production and ammonia-rich wastewater purification [18, 19]. Secondly, the charge storage capacity needed for the redox mediator was underestimated, and the H_2_ evolution period in the cathodic cell was thus not long enough, which complicated the operating conditions.

This issue motivates us to seek out a high-capacity redox system that could persistently and efficiently mediate the ion exchange between anode and cathode during the gas evolution process. In view of their high power density, fast charge–discharge rate and long cycling life, pseudocapacitors as electrochemical energy storage media have great application as mediators for decoupling water electrolysis [20, 21]. Besides, the alkaline hydrogen evolution reaction (HER), which involves the dissociation of water molecules with the generation of H* (H_2_O + e^−^ = H* + OH^−^), has a high activation barrier due to the sluggish reaction kinetics [22, 23]. Thus, the development of dual functional catalysts that exhibit highly efficient HER and superior capacity is of key interest and a significant innovation.

Transition metal phosphides (TMPs), with high thermal stability and a broad array of chemical structures, have been identified as potential catalysts for electrocatalysis and energy storage [24, 25]. Specifically, modderite structure CoP, with a stable orthorhombic crystal structure (space group: Pnma) where Co^3+^ is bonded to six equivalent P^3−^ atoms to form corner-sharing CoP_6_ octahedra, possesses high reactivity but inferior electronic conductivity [26, 27]. To solve the problem, it is widely accepted that hybridization with highly conductive species is a feasible strategy to tune the valence electron state, thereby benefiting the electrical conductivity and electrochemical reaction kinetics [28, 29]. Based on the above considerations, we herein successfully developed a doping-assisted approach to obtain metallic Co_2_P/metalloid CoP nanoarrays (denoted as Co_2_P/CoP NAs) as bifunctional electrocatalysts for charge storage and hydrogen generation. Hierarchical array structures were designed with increased surface area to facilitate diffusion of electrolyte into the electrode during the electrocatalytic process. The as-prepared Co_2_P/CoP NAs electrode could deliver a large specific areal capacitance of 10.52 F cm^−2^ and low HER overpotential of 40 mV at a current density of 10 mA cm^−2^ in an alkaline electrolyte. Theoretical calculations were carried out to better understand the effect of Co_2_P incorporation on the enhancement of electrocatalytic activity. In the water electrolyzer, the high capacitance of the Co_2_P/CoP NAs mediator supported persistent H_2_ separation, where the H_2_ production period lasted for 1500 s at a current density of 10 mA cm^−2^, almost 3.1 times longer than that for pure CoP (480 s). Alternatively, the energy stored in the mediator could be exhausted via coupling with the oxidation of ammonia, with NiSe as the anode (Fig. 1c). The shortened oxidation path at the anode was responsible for enhanced charge transfer, whereby a total driving voltage of 1.55 V was required to support a current density of 10 mA cm^−2^ in the ammonia-containing solution, ~ 0.14 V lower than that under alkaline conditions.

## Experimental Section

### Reagents and Materials

Cobalt acetate tetrahydrate (Co(CH_3_COO)_2_·4H_2_O), ammonium fluoride (NH_4_F), urea (CO(NH_2_)_2_), selenium (Se) and dimethylformamide (C_3_H_7_NO) were purchased from Sinopharm Chemical Reagents Beijing Co., Ltd (China). Zinc nitrate hexahydrate (Zn(NO_3_)_2_·6H_2_O), sodium hypophosphite monohydrate (NaH_2_PO_2_·H_2_O), potassium hydroxide (KOH) were purchased from Sigma-Aldrich. Milli-Q ultrapure water was used for all experiments. All of the reagents were analytical grade and used without further purification.

### Synthesis of Zn–Co Hydroxide Nanoarrays

Cobalt foam (CF) was cleaned in acetone (15 min), 0.1 M hydrochloric acid (15 min) and ethanol (15 min) by sequential ultrasonication. Zn–Co hydroxide arrays were prepared on CFs via a hydrothermal method. 4 mmol Zn(NO_3_)_2_·6H_2_O and Co(CH_3_COO)_2_·4H_2_O with molar ratio of 1:2 were dissolved in 40 mL of distilled water at room temperature. Then, 8 mmol of NH_4_F and 10 mmol of CO(NH_2_)_2_ were added into the mixture under vigorous stirring to form a clear solution. After immersing the cleaned CF (1 × 4 cm^2^) in the homogeneous solution, the autoclave was sealed and maintained at 100 °C for 6 h and then cooled down to room temperature naturally.

### Phosphorization of Zn–Co Hydroxide Nanoarrays

The Zn–Co hydroxide nanoarray-loaded electrode and NaH_2_PO_2_ were located in different positions in a quartz boat, with NaH_2_PO_2_ at the upstream side of the furnace. Subsequently, the sample was heated to 400 °C at a ramp rate of 10 °C min^−1^ and maintained for 120 min in an Ar atmosphere. The furnace was naturally cooled down to room temperature under Ar atmosphere.

### Acid Etching of Phosphorized Zn–Co Nanoarrays

The phosphorized Zn–Co nanoarrays were immersed into a 0.1 M HCl solution and stirred for 6 h. After that, the sample was taken out and washed, which was denoted as Co_2_P/CoP NAs. For comparison, pure CoP on CF was prepared without the addition of Zn(NO_3_)_2_·6H_2_O.

### Preparation of NiSe Electrode

Nickel foam (NF) was sequentially washed using acetone, 2.0 M HCl, and a mixture of deionized water and absolute ethanol for 10 min under ultrasonication, respectively. After that, 2.5 mmol Se, 7.5 mmol NaOH, 0.14 mL hydrazine and 25 mL dimethylformamide (DMF) were dissolved in 40 mL deionized water under vigorous stirring. The NF was immersed in the solution and transferred into a 50-mL Teflon-lined stainless steel autoclave at 180 °C for 1 h. The as-obtained NiSe/NF was washed with deionized water several times.

### Structural Characterization

The crystal structure of the samples was characterized by powder X-ray diffraction (XRD) (PANalytical Inc.) using Cu Kα radiation with a fixed slit. The morphology and information on the lattice spacing of the materials were obtained using a field emission scanning electron microscope (FE-SEM, Hitachi, Japan) and high-resolution transmission electron microscope (HRTEM) equipped with an X-ray energy-dispersive spectrometer (EDS) (JEM-2100F, JEOL, Japan). X-ray photoelectron spectroscopy (XPS) analyses were carried out with a PHI5000 Versa Probe system. All the spectra were referenced to the C 1 s binding energy (BE) of 284.8 eV. The specific surface area was measured by N_2_ adsorption–desorption isotherms at 77 K using the Brunauer–Emmett–Teller (BET, ASAP2460, Micromeritics) method. Raman spectra were obtained using a confocal Raman microscope (Renishaw, England). In situ X-ray absorption spectra (XAS) at the Co K-edge were recorded at beam line BL14W1 of Shanghai Synchrotron Radiation Facility (SSRF), China.

### Electrochemical Characterization

Electrochemical measurements were carried out with an electrochemical workstation (Gamry) using a standard three-electrode setup in 1.0 M KOH electrolytes. The as-synthesized catalytic electrodes were used as working electrodes and a graphite rod and Ag/AgCl as counter electrode and reference electrode, respectively. The electrochemical profiles of electrodes were investigated using linear sweep voltammetry (LSV), cyclic voltammetry (CV) and galvanostatic charge–discharge measurements, respectively. All potentials were quoted with respect to the reversible hydrogen electrode (RHE) according to equation: *E*_RHE_ = *E*_Ag/AgCl_ + 0.059 × pH + 0.2. Unless specifically noted, all of the potentials are given without iR compensation. Cyclic voltammetry (CV) measurements were applied to probe the electrochemical double-layer capacitance (*C*_dl_) in the non-Faradaic region for estimating the effective electrochemical surface area (ECSA). Electrochemical impedance spectroscopy (EIS) measurements were carried out using this apparatus over a frequency range of 100 kHz to 0.01 Hz with AC amplitude of 5 mV. The areal capacitance C (F cm^−2^) of the electrode can be calculated from the galvanostatic charge–discharge curves based on the following equation: *C* = (*I* × ∆*t*)/(*A* × ∆*V*), where I is the discharge current (A), Δ*t* is the discharge time (s), *A* is the area of electrode (cm^2^) and Δ*V* is potential change during discharge (V).

### Water Electrolysis Investigation

A water electrolysis system was constructed with NiSe/NF, Co_2_P/CoP NAs and a Co-based mediator electrode as the anode, cathode and mediator, respectively. Water electrolysis was investigated using chronopotentiometry measurements with applied currents of 10 and 20 mA cm^−2^. Step 1 was performed in 1.0 M KOH electrolyte, where the HER electrode and Co_2_P/CoP NAs electrode were connected to the cathode and anode of a DC power supply. Step 2 was started after finishing the charging process of the redox mediator. In Step 2, the high-valent cobalt electrode and NiSe/NF electrode were connected to the cathode and anode of the DC power supply for electrolysis. Step 2 automatically stopped when the discharge of the Co-based electrode finished. Cell voltages (voltage vs. time) of Steps 1 and 2 were recorded to characterize the electrolysis profile. With the use of an additional Ag/AgCl reference electrode, the chronopotentiometry data (potential vs. time) of the Co-based mediator electrode were recorded in Steps 1 and 2.

### Density Functional Theory Calculation

The CoP (111) and CoP(111)/Co_2_P(111) interface were built, and the vacuum space along the z direction was set to be 15 Å, which is enough to avoid interaction between two neighboring images. H, H_2_O and H–OH groups were absorbed on the surface of these materials. First principles calculations were carried out in the framework of density functional theory, including structural and electronic aspects, based on the Cambridge Sequential Total Energy Package known as CASTEP [30]. The exchange–correlation functional under the generalized gradient approximation (GGA) with norm-conserving pseudopotentials and Perdew–Burke–Ernzerhof functional were adopted to describe the electron–electron interaction [31]. An energy cutoff of 750 eV was used, and a k-point sampling set of 5 × 5 × 1 was tested to convergence. A force tolerance of 0.01 eV Å^−1^, energy tolerance of 5.0 × 10^−7^eV per atom and maximum displacement of 5.0 × 10^−4^ Å were considered. Each atom in the storage models was allowed to relax to the minimum enthalpy without any constraints. The transition state of the H_2_O → H–OH process was calculated. Additionally, the complete linear synchronous transit (LST)/quadratic synchronous transit (QST) search protocol was used, and the root mean square (RMS) convergence of 0.05 eV Å was set for transition states (TS). The adsorption energy Δ*E* of A groups on the surface of substrates was defined as: Δ*E* = E_*A_ − (E_*_ + *E*_A_), where *A and * denote the adsorption of A groups on substrates and the bare substrates and *E*_A_ denotes the energy of A groups. The free energy change Δ*G* of the reaction was calculated as the difference between the free energies of the initial and final states as shown below: Δ*G* = Δ*E* + ΔZPE − *T*Δ*S,* where *E* is the energy calculated by DFT, *ZPE* is the zero point energy and *S* denotes the entropy.

## Results and Discussion

### Synthesis and Characterization of Co_2_P/CoP NAs

Figure 1d shows the fabrication procedure of Co_2_P/CoP NAs on Co foam (CF). By using a bottom-up hydrothermal process, a Zn–Co hydroxide arrayed structure was first synthesized on macroporous CF (Fig. S1). Briefly, Zn^2+^ with lower solubility preferentially precipitated in solution compared with Co hydroxide [32, 33]. Thus, in the competitive co-precipitation process, Zn hydroxides were first formed as the “trunk” on the CF. As the Zn^2+^ concentration decreased, Co hydroxide nanosheets successively precipitated on the Zn-rich trunks, resulting in a Zn–Co hierarchical structure (Fig. S2a). After phosphorization, the Zn–Co hydroxide was transformed into phosphide, while the arrayed structure was well preserved (Fig. S2b). The hierarchical Co_2_P/CoP NAs could be finally achieved by an etching approach using 0.1 M HCl solution.

**Fig. 2:**
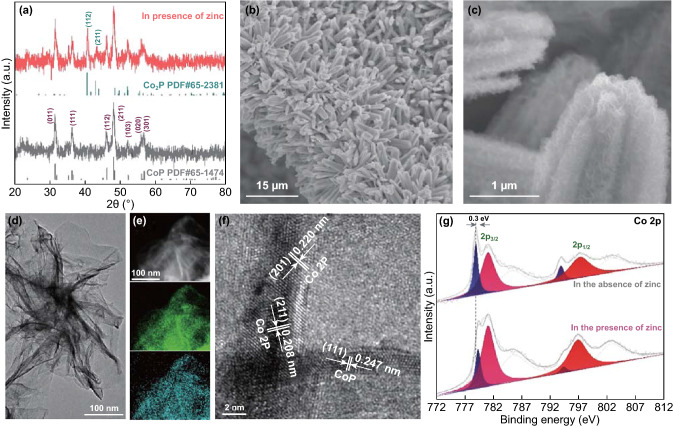
**a** X-ray diffraction patterns of pure CoP and Co_2_P/CoP NAs. **b**, **c** SEM images of Co_2_P/CoP NAs-loaded electrode. **d** TEM images of Co_2_P/CoP NAs sheets with STEM-EDS mapping in **e**. **f** HRTEM images of Co_2_P/CoP NAs. **g** High-resolution Co 2p XPS spectrum of pure CoP and Co_2_P/CoP NAs

The crystal structures of the as-prepared products were subsequently investigated by XRD. In the absence of zinc, the orthorhombic crystal structure of CoP was well indexed to a reference XRD pattern (ICDD PDF: 65-1474), whereas mixed crystals were achieved with Zn addition, exhibiting orthorhombic CoP and Co_2_P structures (ICDD PDF: 65-2381) (Fig. 2a). As shown by SEM in Fig. 2b, c, the hierarchical Co_2_P/CoP NAs were composed of numerous fuzzy flakes, which was in sharp contrast to the irregular morphology observed in the absence of Zn^2+^ (Fig. S3a, b). A two-dimensional nanoflake morphology with a thickness of several layers, which was beneficial for the exposure of catalytically active sites, was further confirmed by TEM images (Fig. 2d). Meanwhile, EDX elemental mapping revealed that Co and P were homogeneously distributed on the surface of the nanosheets (Fig. 2e). In good agreement with the XRD analysis, the high-resolution TEM (HRTEM) image showed that the Co_2_P phase was well preserved in the CoP nanosheets (Fig. 2f). The lattice spacing of 0.247 nm was assigned to the (111) plane of orthorhombic CoP, while lattice distances of 0.208 and 0.220 nm corresponded to the (211) and (201) planes of Co_2_P, respectively. Analogously, the pure CoP exhibited the crystal phase of CoP under HRTEM observation (Fig. S3c–e). Additionally, the Brunauer–Emmett–Teller (BET) surface area of porous Co_2_P/CoP NAs (20.25 m^2^ g^−1^) was 4 times higher than that for CoP (5.4 m^2^ g^−1^) (Fig. S4). To probe the formation mechanism of the Co_2_P/CoP structure, various Zn amount and annealing temperatures were investigated. As shown in Fig. S5, the Co_2_P phase could be observed in the presence of Zn and the corresponding peaks became narrow and sharp with the increase in the amount of Zn. Notably, only CoP could be synthesized under 300 °C pyrolysis conditions in the presence of Zn (Fig. S6). The control of the amount of Zn and the annealing temperature suggested the critical roles of Zn content and phosphorization temperature in adjusting the Co_2_P/CoP hierarchical structure (Figs. S5, S6). XPS measurements were employed to probe the surface chemistry of the fabricated phosphides. The XPS survey scan spectrum of Co_2_P/CoP NAs implied the presence of Zn, Co and P elements (Fig. S7). In the high-resolution Co 2p region, the peak area at the position of 779.0 eV assigned to Co 2p_3/2_ was rapidly decreased after incorporation of Co_2_P, suggesting the increased electron density of partial positively charged Co species (Co^δ+^) in the mixed crystal phase (Fig. 2g) [34]. It was noted that the incorporation of Co_2_P led to a shift of Co^δ+^ to higher energy levels by 0.3 eV and a negative shift of P 2*p*_3/2_ by 0.2 eV, indicating strong electronic interactions between Co and P (Figs. 2g and S7c) [35].

### Investigation of Electrochemical Charge Storage and HER Activities

**Fig. 3:**
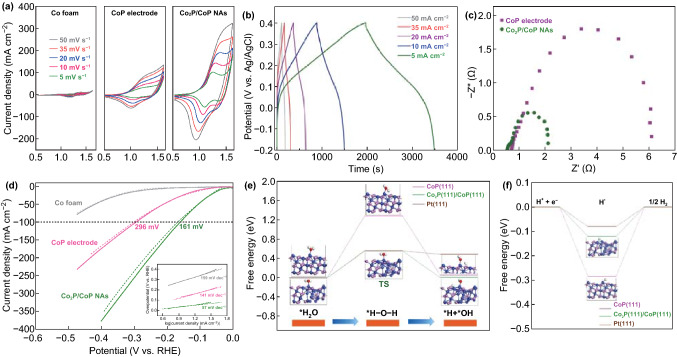
**a** CV curves of blank CF, pure CoP and Co_2_P/CoP NAs electrodes at different scan rates in 1 M KOH electrolyte. **b** Charge/discharge curves of Co_2_P/CoP NAs at various current densities. **c** Nyquist impedance spectra of pure CoP and Co_2_P/CoP NAs electrodes. **d** LSV curves for the Co-based electrodes for the H_2_ evolution at a scan rate of 2 mV s^−1^ in 1 M KOH solution (thick line) and ammonia-containing solution (fine line). Calculated adsorption free energy diagram for **e** water dissociation and **f** H adsorption potential on pure CoP (111), Co_2_P(111)/CoP(111) and Pt (111), respectively. Blue balls: Ni; pink balls: P; red balls: O. (Color figure online)

The capacitive behavior of the hierarchical Co_2_P/CoP NAs material was investigated in a three-electrode cell using cyclic voltammetry (CV) measurements. Figure 3a presents the typical CV curves of as-prepared catalysts in 1.0 M KOH electrolyte at different scan rates between 0.5 and 1.6 V versus RHE. A redox peak can be clearly observed from the CV curves of the Co-based materials, which is ascribed to the reversible Faradaic process of interconversion of Co(II)/Co(III) and Co(III)/Co(IV) couples. The similarity of the Co 2p spectrum with that of standard Co_3_O_4_ and the disappearance of metal phosphide after long-term tests suggested the oxidization of Co atoms to form CoO_*x*_ species (Fig. S8a, b). The oxidation phenomenon was also revealed by the increased O amount, probably implying that the essential active sites of Co_2_P/CoP NAs for the electrochemical energy storage were metal oxide/hydroxides formed on the surface (Fig. S8c, d). For Co_2_P/CoP electrode materials, surface Faradaic reactions could thus be proposed in alkaline medium as follows [36, 37]:

1 $${\text{Co}}^{\delta + } + 2{\text{OH}}^{ - } \to {\text{Co}}^{\text{II}} \left( {\text{OH}} \right)_{2} + (2 - \delta ){\text{e}}^{ - }$$

2 $${\text{Co}}^{\text{II}} \left( {\text{OH}} \right)_{2} + {\text{OH}}^{ - } \leftrightarrow {\text{Co}}^{\text{III}} {\text{OOH}} + {\text{H}}_{2} {\text{O}} + {\text{e}}^{ - }$$

3 $${\text{Co}}^{\text{III}} {\text{OOH}} + {\text{OH}}^{ - } \leftrightarrow {\text{Co}}^{\text{IV}} {\text{O}}_{2} + {\text{H}}_{2} {\text{O}} + {\text{e}}^{ - }$$

Meanwhile, symmetric peaks can be observed for both anodic and cathodic currents, implying the reversibility of the Co_2_P/CoP-loaded electrode. However, due to the internal resistance, the anodic and cathodic peaks shifted with increasing scan rate. The CV curves of Co foam demonstrated that the current was much lower than that for the Co_2_P/CoP NAs-loaded electrode, indicating that capacitive effects arising from the Co foam substrate can be neglected (Fig. 3a). The CV curve of the Co_2_P/CoP NAs exhibited remarkably higher current in comparison with pure CoP, suggesting that the incorporation of Co_2_P could enhance the charge storage capacity (Fig. 3a).

To further estimate the stable potential window of the as-synthesized catalysts, galvanostatic charging and discharging of the samples were performed in 1 M KOH solution using a saturated Ag/AgCl reference electrode and a graphite rod counter electrode. Figure 3b depicts the charge–discharge curves of Co_2_P/CoP NAs at different current densities (5–50 mA cm^−2^) in the range of − 0.2 and 0.4 V versus Ag/AgCl. In agreement with the CV results, the lack of defined voltage plateaus in the charge/discharge curves suggested the presence of pseudo-capacitive behavior. The areal capacitance of the Co_2_P/CoP NAs electrode slowly decreased as current density increased from 5 to 50 mA cm^−2^. The discharge time for Co_2_P/CoP NAs reached 2,000 s at a current density of 5 mA cm^−2^, resulting in a high areal capacitance of 12.95 F cm^−2^, ~ 4.5 times higher than that for the pure CoP-loaded electrode (2.86 F cm^−2^) (Fig. S9a). The Co_2_P/CoP NAs electrode also exhibited excellent rate capability performance, with 61% capacitance retained at the high current density of 50 mA cm^−2^ (7.9 F cm^−2^) in comparison with 5 mA cm^−2^ (Fig. S9b). The capacitive performance of the Co_2_P/CoP NAs regulated by the Zn content, and annealing temperature was also evaluated and is shown in Fig. S10. As expected, the sample obtained with the molar ratio of Co to Zn (2 to 1) and annealing temperature of 400 °C possessed the optimal capacitance activity. The extremely low amount of Zn identified by EDX spectroscopy, and inductively coupled plasma mass spectrometry (ICP–MS) suggested its insufficiency to alter the capacitance activity (Fig. S11). To provide further insights into the superior charge storage, the physical origins of the electrode kinetics were further examined via electrochemical impedance spectroscopy (EIS). A small semicircle in the high-frequency region reflects low charge transfer resistance [38, 39]. The Co_2_P/CoP NAs electrode exhibits a smaller semicircle diameter than that of pure CoP, suggesting that the introduction of Co_2_P could lower charge transfer resistance and accelerate electron transport across the interface between Co_2_P/CoP NAs and electrolytes (Fig. 3c). The synergistic effect between Co_2_P and CoP, where the CoP is responsible for high reactivity while Co_2_P accounts for electronic conductivity, as well as the porous arrayed structure that benefits the high diffusivity of ions in the electrolyte, could play a vital role in boosting the charge storage.

To construct an electrolyzer for electrocatalytic H_2_ evolution, an active HER catalyst was simultaneously needed. Fortunately, the developed Co_2_P/CoP NAs also exhibited the highest catalytic activity toward HER, with low overpotential of 160 mV at 100 mA cm^−2^ and Tafel slope of 57 mV dec^−1^ (Figs. 3d and S12). The electrocatalytic performance of Co_2_P/CoP NAs is comparable to those of other highly efficient HER electrocatalysts reported recently (Table S1). To reach the same current density, an overpotential of 296 mV was needed for the pure CoP electrode. In the meantime, the HER polarization curves of the Co-based sample obtained with the addition of chemicals were at similar levels to that obtained in basic conditions, indicating the presence of robust catalytic activity under different conditions. We next investigated the effect of the electrochemically active surface area (ECSA) on HER performance, which was estimated by measuring the electrochemical double-layer capacitance (*C*_dl_). The *C*_dl_ value of Co_2_P/CoP NAs was evaluated by CVs to be 8.1 mF cm^−2^, which is larger than that of CoP (3.7 mF cm^−2^), demonstrating the greater exposure of catalytically active sites (Fig. S13). The EIS results suggested superior charge transfer performance between the surface of the Co_2_P/CoP NAs electrode and the electrolyte (Fig. S14). The long-term stability of the electrode was also investigated at current densities of 10 and 20 mA cm^−2^ (Fig. S15a). The Co_2_P/CoP NAs electrode retained steady activity, and no noticeable overpotential augment was observed after more than 28 h of H_2_ release. Moreover, the LSV curve recorded after 2000 cycles almost overlapped the initial one (Fig. S15b). After durability tests, the electrode was subjected to post-characterizations to investigate the changes in the morphology and chemical structure of the Co_2_P/CoP NAs electrocatalyst. The electronic patterns directly show that the structure of vertically aligned nanorod arrays and the clear lattice spacing were well maintained after the HER stability test (Fig. S16d–f). Meanwhile, XRD and XPS results demonstrated that the crystal structure and chemical composition remain nearly unchanged after HER, exhibiting the structural robustness of the catalysts (Fig. S16a–c).

To understand the origin of the high HER activity of Co_2_P/CoP NAs, theoretical investigations were conducted based on DFT calculations. Figure S17 exhibits the schematic models of the matched Co_2_P(111)/CoP(111) heterostructure and pure CoP(111), respectively. The H_2_O molecule has a lower adsorption energy of − 1.76 eV on Co_2_P(111)/CoP(111) than that on CoP(111) (− 1.68 eV), indicating more favorable H_2_O adsorption for Co_2_P(111)/CoP(111) (Fig. S18). Importantly, the water dissociation on Co_2_P(111)/CoP(111) experiences a quite low barrier of 0.57 eV, more promising than that on CoP(111) (1.29 eV), which is even comparable to that of the Pt (111) (0.563 eV) (Fig. 3e) [40]. Meanwhile, the lower absolute values of Δ*G*_H*_ enable a suitable H* adsorption strength [41]. Figure 3f presents that the CoP (111) surface has a Δ*G*_H*_ value of − 0.28 eV on the Co site. After the incorporation of Co_2_P, the Δ*G*_H*_ increased to − 0.12 eV on the Co–Co bridge site, which is more thermo-neutral than that of pure CoP and close to that of the Pt (111) (− 0.08 eV) (Figs. 3f and S19) [40, 42]. Additionally, a reduced valance charge for Co near the Co_2_P(111)/CoP(111) interface can be clearly observed, consistent with the above XPS results, which is beneficial for hydrogen desorption from Co sites (Co_2_P(111)/CoP(111)) (Fig. S20 and Table S2). From the above analysis, it was deduced that the Co sites near Co_2_P/CoP interfaces served as the actual active sites for HER.

### Performance of the Two-Step Alkaline Water Electrolysis

**Fig. 4:**
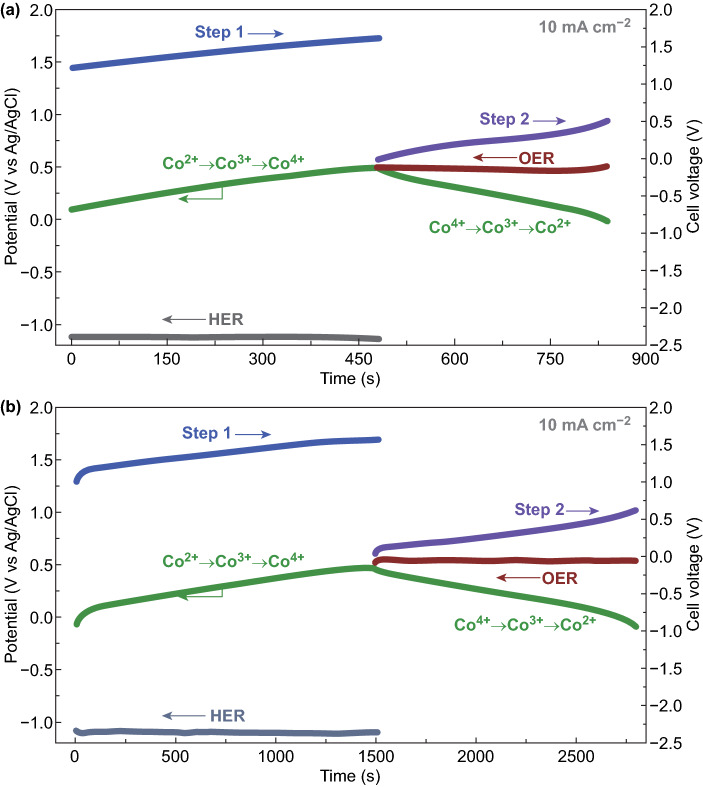
Chronopotentiometry data (potential vs. time) of batch reactor using **a** pure CoP or **b** Co_2_P/CoP NAs electrode. Chronopotentiometry curves were recorded at a current density of 10 mA cm^−2^. [(Voltage of Step 1) = (potential of Co charge)—(potential of HER); (voltage of Step 2) = (potential of OER)—(Potential of Co discharge)]. Voltages for H_2_ production (Step 1) and O_2_ production (Step 2) are labeled using the blue and purple lines, respectively. Chronopotentiometry data (potential vs time) of HER electrode, mediator electrode and OER electrode are labeled using the gray, green and crimson lines, respectively. (Color figure online)

In the meantime, a Ni^II^ → Ni^IV^ earth-abundant catalyst was applied as the catalyst for the anodic reaction, aiming for a low bias voltage to achieve high H_2_ production efficiency. Based on the combined results of XRD, TEM-EDS, and XPS analysis, nickel selenide (NiSe) was shown to form a good coating on the Ni foam (NF) (Fig. S21). As observed from the polarization curve, the NiSe/NF electrode can support a current density of 50 mA cm^−2^ at a potential of 1.65 V for OER (Fig. S22). For better implementation, a batch-type reactor for decoupling the conventional water electrolytic process was constructed using NiSe electrodes as anode, Co_2_P/CoP NAs-loaded electrodes as cathode and Co-based electrodes as redox mediator. Water electrolysis in the cell was investigated by chronopotentiometry measurements with a current density of 10 mA cm^−2^. As depicted in Fig. 4a, the chronopotentiometry data of the anode (anodic potential vs. time) and cathode (cathodic potential vs. time) were investigated during the electrolysis process using pure CoP as the redox mediator. Two steps (Steps 1 and 2) with different cell voltages were involved in the electrolysis process. In the hydrogen cell (Step 1), HER occurs at the cathode, and OH^−^ ions are consumed by the mediator, transforming Co(II) to Co(IV). Step 1 exhibits a cell voltage of ~ 1.45 V, which is derived from the difference between the anodic potential of Co oxidation and cathodic potential of H_2_O reduction (Fig. S23a). In the oxygen cell (Step 2), OER occurs at the anode, while the NiSe/NF electrode interacts with OH^−^ to evolve O_2_, and the cathodic reaction synchronously occurring in the counter compartment involves reduction of Co(IV) to Co(II), thus accomplishing the whole regeneration cycle. In the O_2_-production process, the cell voltage is ~ 0.25 V, which is calculated from the potential difference between the anodic oxidation of OH^−^ and the cathodic reduction of Co(IV) to Co(II) (Fig. S23a). In this configuration, the cell voltage for step 1 markedly increases after 480 s, suggesting that Co(II) has been completely converted to Co(IV). Subsequently, the electrolysis in step 2 was automatically completed after 357 s.

It should be noted that the hierarchical Co_2_P/CoP NAs material, as a well-engineered electrode, exhibited much higher energy storage efficiency and longer cycle life than pure CoP. These characteristics are of great importance to prolong the electrolysis time in each step. To demonstrate this, a batch-type reactor was reconstructed using the hierarchical Co_2_P/CoP NAs material as redox mediator. Under a current density of 10 mA cm^−2^, the H_2_ production time in this alkaline electrolytic cell could be increased to ~ 1500 s, ~ 3.1 times longer than that using the pure CoP electrode (Fig. 4b). At the same time, an unequal electrolysis time (1290 s) for O_2_ production was observed in step 2, which resulted from the rapid release of energy storage capacity in the arrayed nanostructure. Photographic profiles of the H_2_ generation in Step 1 and O_2_ generation in Step 2 are shown in Fig. S24 to further characterize the separated steps. Moreover, the electrolysis process was also investigated at a current density of 20 mA cm^−2^. As depicted in Fig. S25, the stable H_2_ production indicated the flexibility of the system.

### Evaluation of the Two-Step Alkaline Ammonia Electrolysis

Furthermore, the total driving voltage of the two-step alkaline water electrolysis process (Step 1 + Step 2) was calculated according to a previous method [43]. A total driving voltage of 1.69 V could support a current density of 10 mA cm^−2^ in two-step water electrolysis, which was mainly restricted by the sluggish kinetics of anodic OER (Fig. S23b). The question arises, how to strengthen the charge transfer efficiency at the anode in order to decrease the total driving voltage? It has been revealed that the charge transfer at the anode can be efficiently intensified by substitution of reactions of easily oxidized contaminants for OER. As a typical pollutant present in landfill leachate worldwide, aqueous ammonia nitrogen conversion into nitrogen gas should be an environmentally friendly pathway [44, 45]. Thus, a platform for the anodic conversion of ammonia was herein constructed to decrease the total driving voltage via enhancing the anodic charge transfer.

**Fig. 5:**
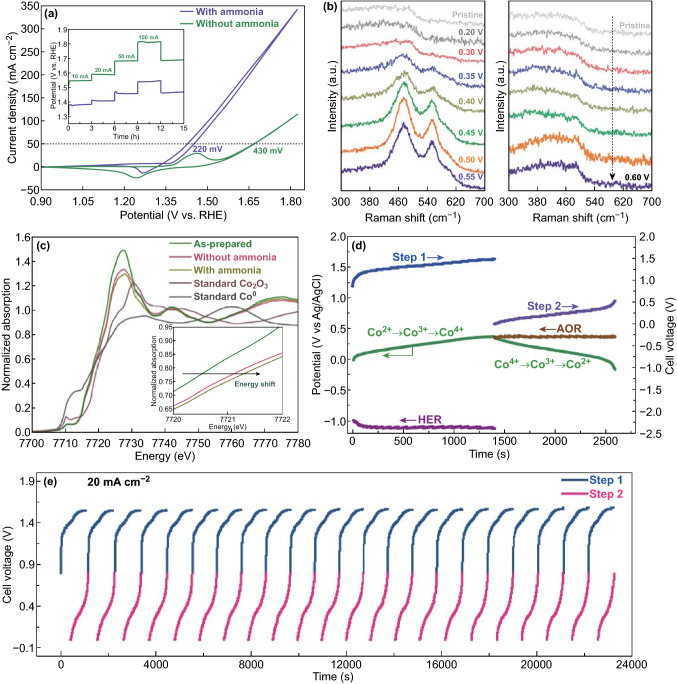
**a** CV curves of NiSe in 1.0 M KOH with presence/absence of ammonia. (Multistep chronopotentiometric curves of NiSe/NF at different current densities are shown as insets.) **b** Raman spectra of the NiSe/NF as a function of potential in basic condition (left) and ammonia-containing solution (right). **c** In situ Co K-edge XANES for Co_2_P/CoP NAs electrode recorded in various electrolytes. Experiments were carried out during potentiostatic process at the potential of 0.45 V versus Ag/AgCl. **d** Chronopotentiometry curve for reactor at a current density of 10 mA cm^−2^, where the voltages for H_2_ production (Step 1) and N_2_ production (Step 2) are labeled by the blue and purple lines, respectively. Chronopotentiometry data (potential vs. time) of the HER electrode (crimson line), Co_2_P/CoP NAs electrode (green line) and AOR electrode (brown line) are provided. AOR denotes ammonia oxidation reaction. **e** Chronopotentiometry curves of H_2_/N_2_ generation cycle at a current density of 20 mA cm^−2^. Chronopotentiometry data of step 1 (H_2_ generation) and step 2 (N_2_ generation) are labeled with blue and pink lines, respectively. (Color figure online)

As shown in Fig. 5a, NiSe exhibited exceptional ammonia conversion performance, with a potential of only 1.52 V required to drive a current density of 100 mA cm^−2^, much lower than the value of 1.79 V for OER. The multistep chronopotentiometric curve of NiSe demonstrated that the potential of the NiSe-based electrode maintained stability at various current densities, indicating outstanding mass transfer properties and mechanical robustness in the ammonia alkaline electrolyte (inset of Fig. 5a). According to the in situ Raman results, the pair of bands at 481 and 561 cm^−1^ at potentials above 0.40 V was attributed to Ni–O vibrations in NiOOH, whereas the peak for NiOOH disappeared after the introduction of ammonia (Fig. 5b) [46, 47]. This strongly implied that Ni^III^OOH as a reactive species was rapidly consumed in the ammonia oxidation process, whereby anodic charge transfer can be intensified by minimizing the energy consumption required for Ni conversion. The phenomenon could also be revealed by the high-resolution XPS spectra of Ni and Se, and EDX element mapping after long-term activation (Fig. S26). By contrast, Raman results for the Co-based electrodes after reaction indicated that the Co^III^ = O vibrational mode could be preserved in the two electrolytes (Fig. S27) [48, 49]. Furthermore, Co K-edge peaks obtained by in situ XANES measurements of the Co_2_P/CoP NAs electrode after applying a potential of 0.45 V versus Ag/AgCl were almost at the same energy in the two electrolytes, implying that the oxidized proportion of Co could be well maintained (Fig. 5c). These results suggested that the ammonia had only a slight effect on the high specific capacity of the electrode. Correspondingly, in the presence of ammonia, similar areal capacitance was observed via comparison of the integrated CV area and calculated charge–discharge curves obtained in basic conditions (Fig. S28). Notably, the Co_2_P/CoP NAs electrode exhibited weaker oxidation ability toward ammonia in comparison with that of the NiSe electrode (Fig. S29). Thus, the NiSe electrode was chosen as the anode to oxidize ammonia.

After the addition of ammonia to the working chamber, the total driving voltage of the two-step electrolysis cell decreased by 0.14 V under the same current density of 10 mA cm^−2^ (Fig. S30). The calculated voltage of 1.55 V at 10 mA cm^−2^ is comparable with that of other landmark reports on redox mediator systems (Table S3). In addition, owing to the negligible effect of ammonia, the vast majority of high-valent Co on the Co_2_P/CoP NAs surface could be preserved in the separate working step, whereby H_2_ production time in the ammonia-containing electrolytic cell still maintained a level of ~ 1400 s (Fig. 5d). This electrolytic cell exhibited stable H_2_ generation for 20 consecutive cycles at the current density of 20 mA cm^−2^ (Fig. 5e). Furthermore, over 200 consecutive cycles of the Co_2_P/CoP NAs electrode at 50 mA cm^−2^ are shown in Fig. S31 to demonstrate the stability of the charge storage.

## Conclusions

In summary, we report that the bifunctional Co_2_P/CoP NAs electrode can act as a robust and low-cost charge mediator for decoupled water electrolysis under alkaline conditions. Theoretical calculations demonstrated that the incorporation of Co_2_P optimizes the hydrogen adsorption free energy and reduces the water dissociation barrier in an alkaline medium, thereby leading to the outstanding HER performance of Co_2_P/CoP NAs. Importantly, the Co_2_P/CoP NAs electrode also exhibited superior charge storage capability, which could prolong the H_2_ evolution time to 1500 s under a current density of 10 mA cm^−2^. To decrease the energy cost, the energy stored in hierarchical Co_2_P/CoP NAs was selectively coupled with the oxidation of ammonia with NiSe as anode, whereby only 0.21 V was required to maintain the current for 1188 s. The work demonstrates the design of cobalt phosphide nanoarrays with ultrahigh capacitance for use as a charge reservoir for persistent hydrogen liberation from contaminant decomposition, thereby achieving an economical and environmentally friendly route to acquiring high-purity H_2_. More importantly, the configuration presented herein may also be applied as a high-performance device for electrocatalytic gas separation in fields including seawater electrolysis or the chlor-alkali process.

## Electronic supplementary material

Below is the link to the electronic supplementary material.

Supplementary material 1 (PDF 3683 kb)
