# Supplementary material for: Arrayed Cobalt Phosphide Electrocatalyst Achieves Low Energy Consumption and Persistent H2 Liberation from Anodic Chemical Conversion
Source: Nanomicro Lett. 2020 Jul 22;12:154. doi: 10.1007/s40820-020-00486-2 (PMC7770902; doi:10.1007/s40820-020-00486-2)
Supplement: Supplementary file 1 — Supplementary material 1 (PDF 3683 kb) [file 40820_2020_486_MOESM1_ESM.pdf]

Supporting Information for

Arrayed Cobalt Phosphide Electrocatalyst Achieves Low Energy Consumption and Persistent H_2 Liberation from Anodic Chemical Conversion

Kai Zhang[1, 4], Gong Zhang[2], Qinghua Ji[2], Jiuhui Qu[2, 3], Huijuan Liu[1, 2, *]

[1]State Key Laboratory of Environmental Aquatic Chemistry, Research Center for Eco-Environmental Sciences, Chinese Academy of Sciences, Beijing 100085, People's Republic of China

[2]Center for Water and Ecology, State Key Joint Laboratory of Environment Simulation and Pollution Control, School of Environment, Tsinghua University, Beijing 100084, People's Republic of China

[3]Key Laboratory of Drinking Water Science and Technology, Research Center for Eco-Environmental Sciences, Chinese Academy of Sciences, Beijing 100085, People's Republic of China

[4]University of Chinese Academy of Sciences, Beijing 100049, People's Republic of China

*Corresponding author. E-mail: hjliu@rcees.ac.cn or hjliu@tsinghua.edu.cn (Huijuan Liu)

Supplementary Figures

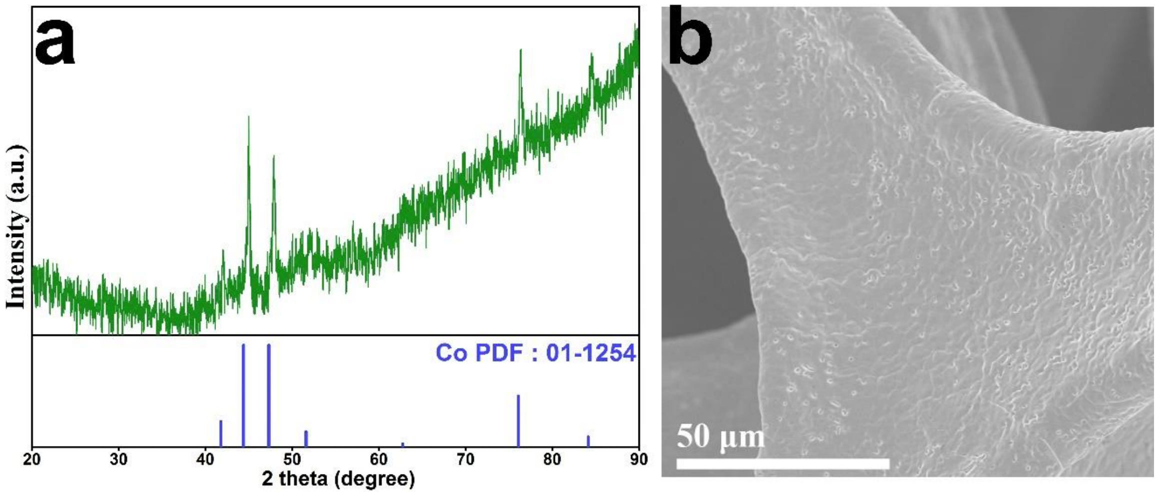

Fig. S1 (**a**) XRD and (**b**) SEM images of pristine Co foam (CF)

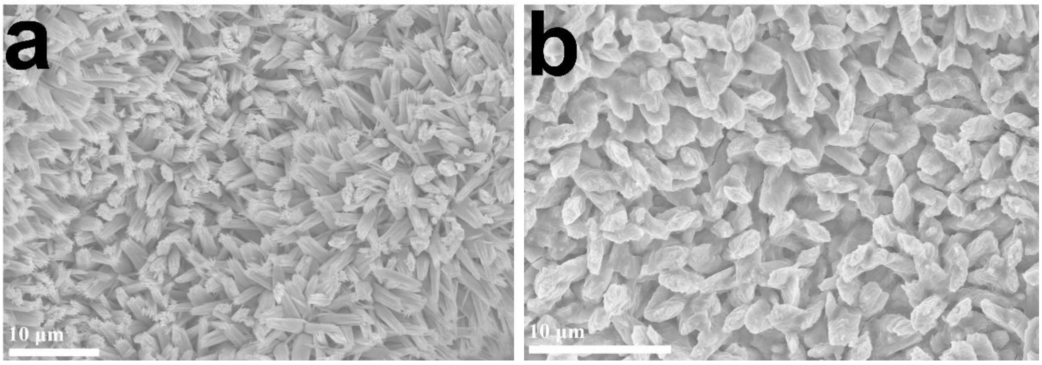

Fig. S2 The SEM images of (**a**) Zn-Co hydroxide nanoarrays and (**b**) phosphatized Zn-Co hydroxide nanoarrays on CF

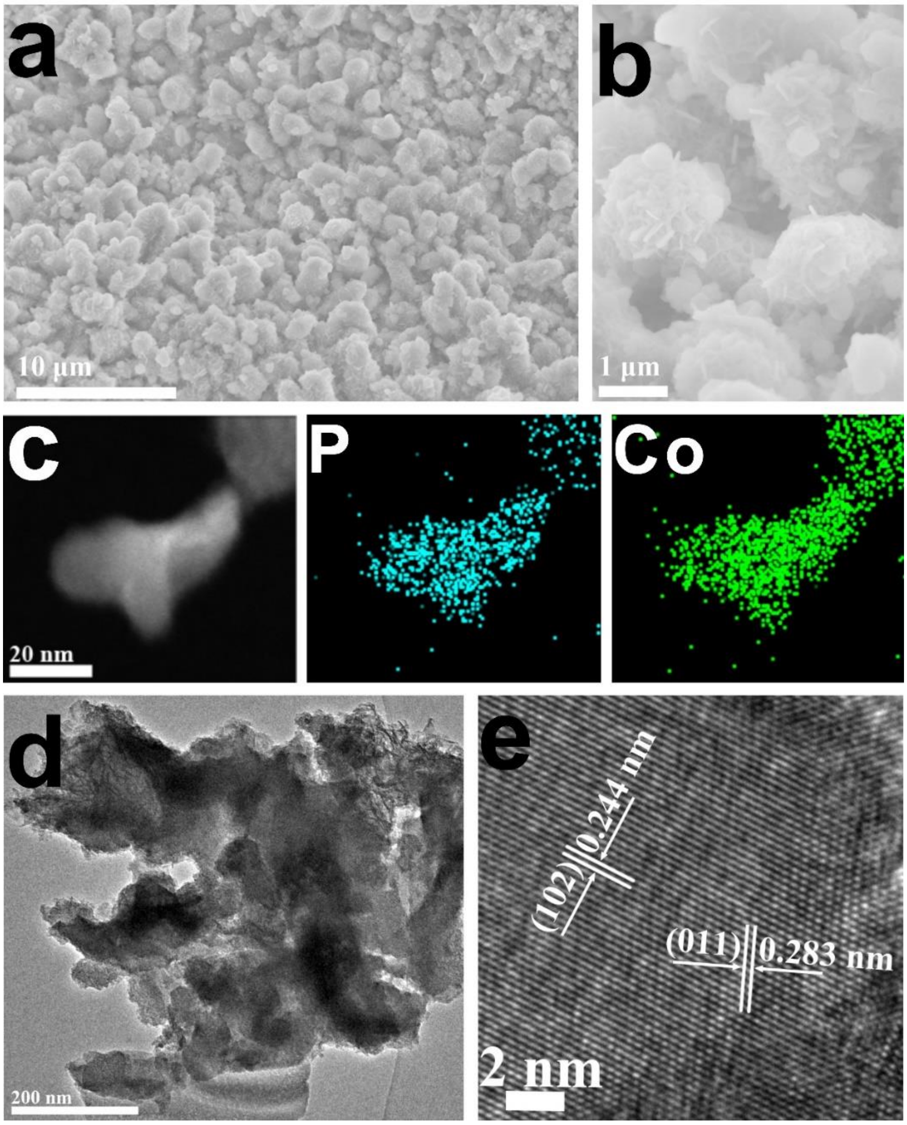

Fig. S3 SEM images of CoP at (**a**) low and (**b**) high magnifications. (**c**) STEM and corresponding elemental mapping of pure CoP. (**d**) TEM and (**e**) HRTEM of pure CoP

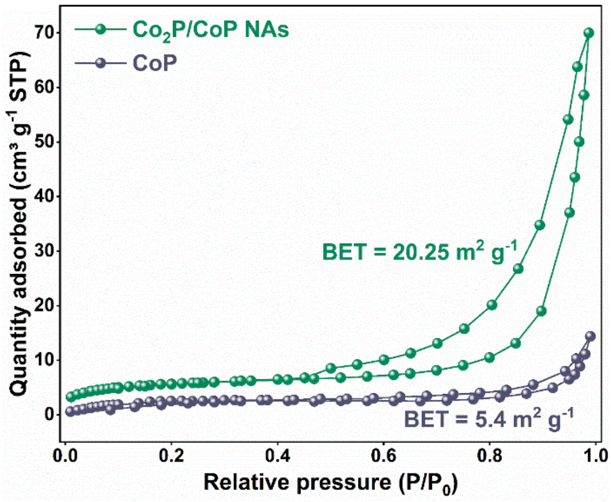

Fig. S4 N_2-adsorption/desorption curves of porous Co_2P/CoP NAs and pure CoP

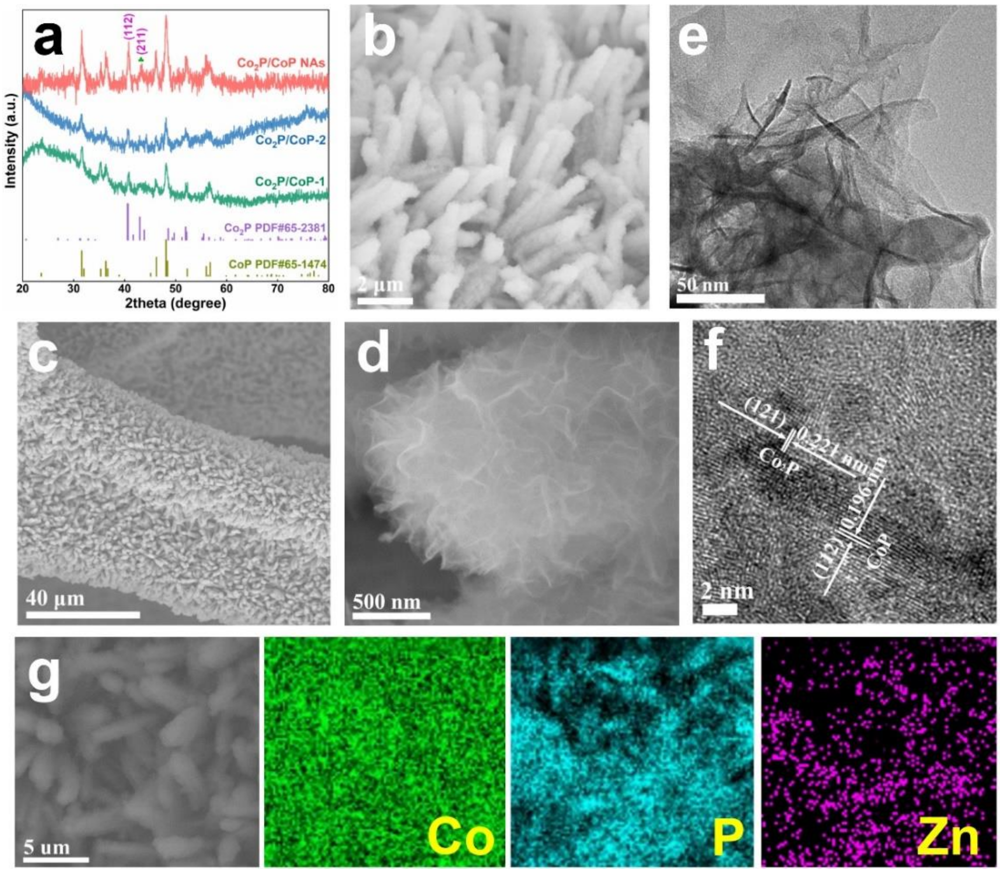

Fig. S5 (**a**) XRD pattern of the as-prepared samples. (**b**) SEM images of the Co_2P/CoP-1 sample. SEM images at (**c**) low and (**d**) high magnifications for the Co_2P/CoP-2 sample. (**e**) TEM and (**f**) HRTEM of the Co_2P/CoP-2 sample. (**g**) The corresponding STEM and elemental mapping of the Co_2P/CoP-2 sample. We prepared the CoP nanoarrays with different molar ratio of Co to Zn (8 to 1 and 4 to 1) using the same method as the synthesis of Co_2P/CoP NAs (molar ratio of Co to Zn (2 to1)). The obtained Co_2P/CoP samples with the molar ratio of Co to Zn (8 to 1 and 4 to 1) are denoted as Co_2P/CoP-1 and Co_2P/CoP-2, respectively

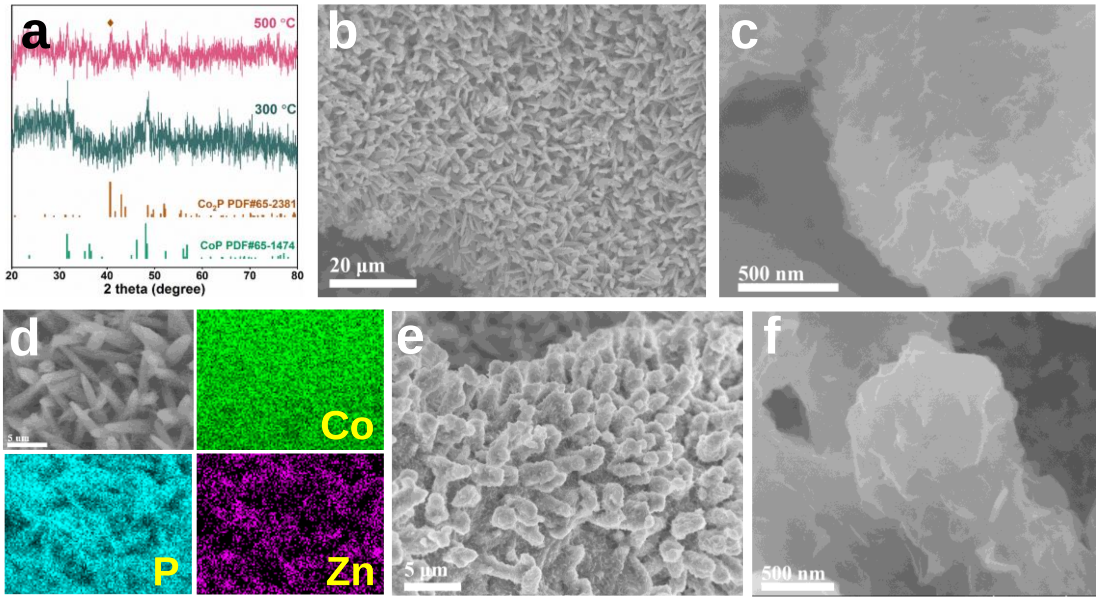

Fig. S6 (**a**) XRD pattern of the as-obtained samples. SEM images at (**b**) low and (**c**) high magnifications for the CoP sample obtained at annealing temperature of 300°C. (**d**) The corresponding STEM and elemental mapping of CoP-300. SEM images at (**e**) low and (**f**) high magnifications for the Co_2P/CoP sample obtained at annealing temperature of 500 °C. We prepared the CoP nanoarrays at different phosphorated temperature (300 and 500 °C) using the same method as the synthesis of Co_2P/CoP NAs

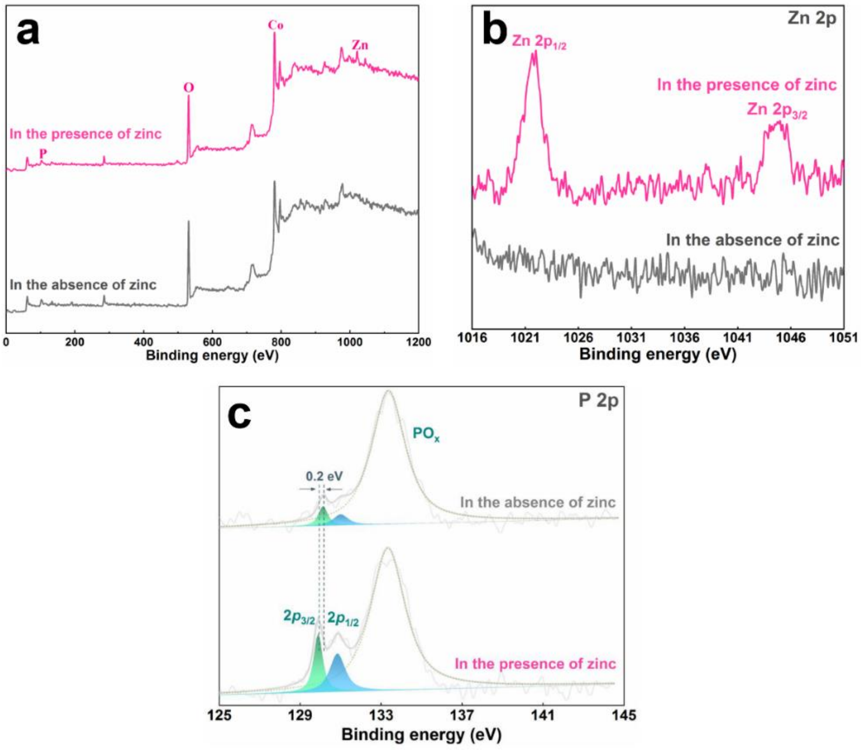

Fig. S7 (**a**) XPS survey scan spectrum and high-resolution XPS spectra of (**b**) Zn and (**c**) P for pure CoP and Co_2P/CoP NAs

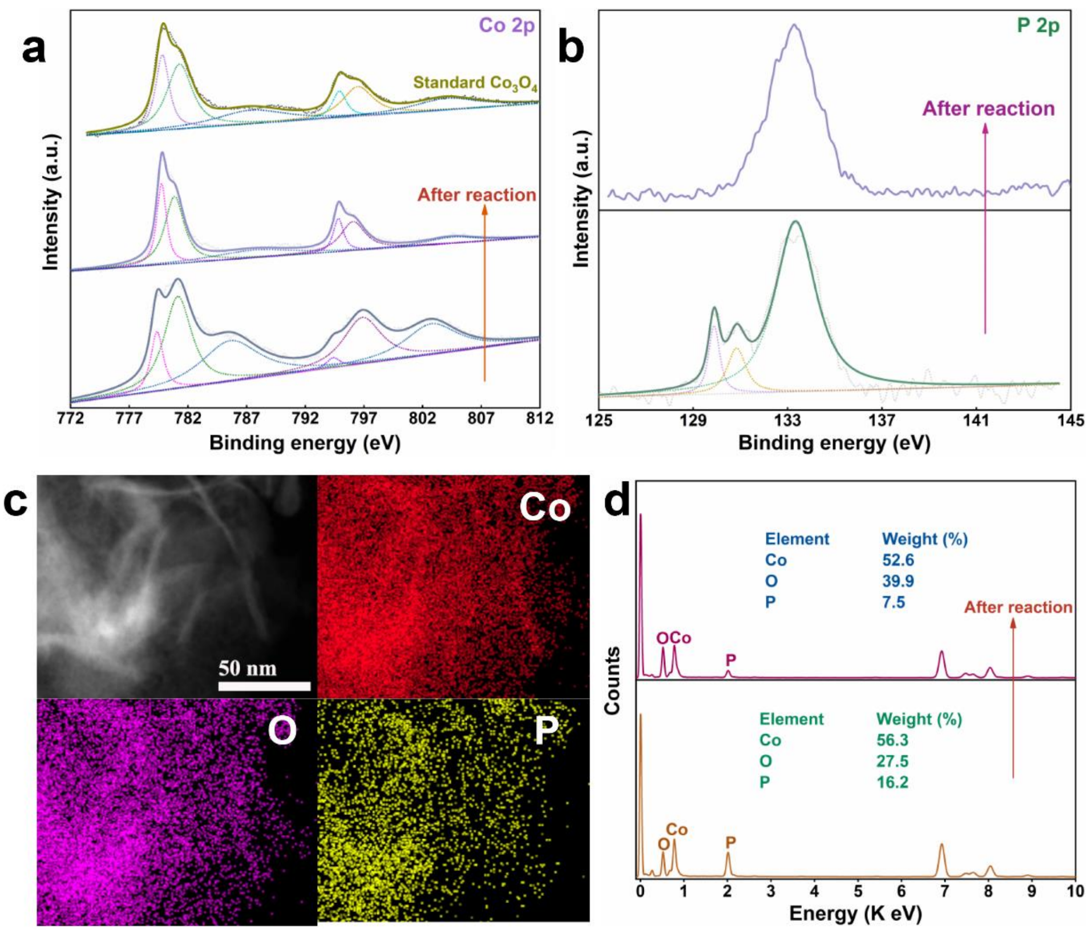

Fig. S8 Structural and morphological characterization of the Co_2P/CoP NAs after long-time reaction. Comparison of High resolution XPS spectra of (**a**) Co and (**b**) P. (**c**) EDX mapping images of Co, O, and P and the corresponding EDX analysis of the Co_2P/CoP NAs after reaction

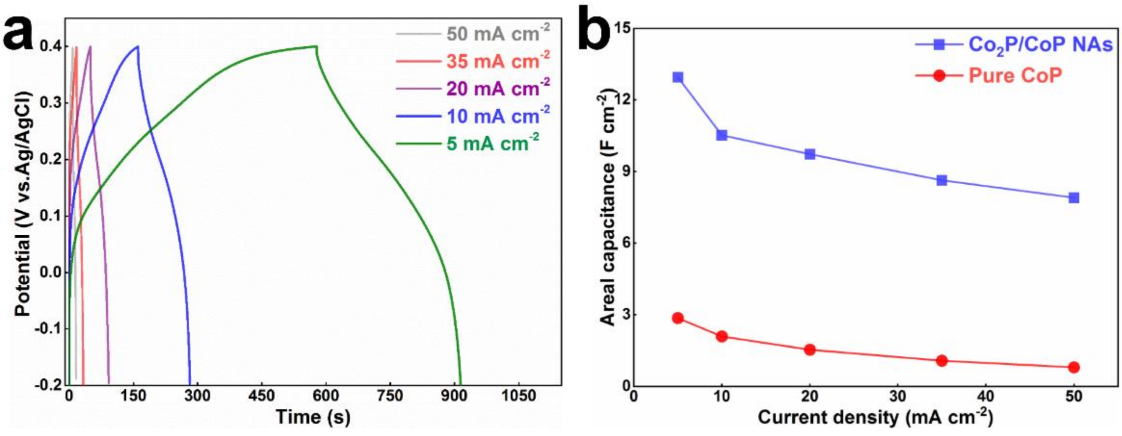

Fig. S9 (**a**) Galvanostatic charge/discharge curves for pure CoP at different current densities in 1.0 M KOH. (**b**) Areal capacitance of pure CoP and Co_2P/CoP NAs as a function of the current densities based on charge/discharge curves

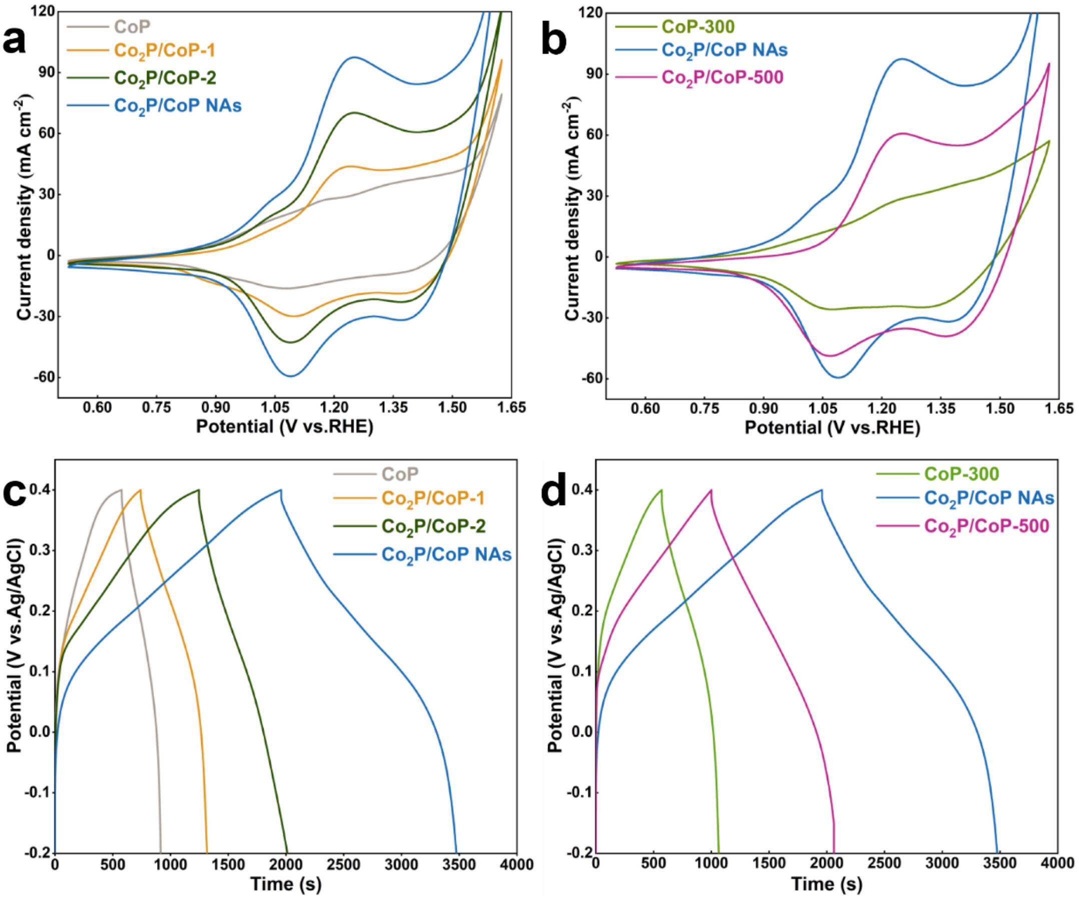

Fig. S10 The CV curves of Co-based samples obtained under (**a**) different ratio of Zn to Co and (**b**) various temperature in 1 M KOH electrolyte at different scan rates. The obtained Co_2P/CoP samples with the molar ratio of Co to Zn (8 to 1 and 4 to 1) are denoted as $Co_2P/CoP-1$ and $Co_2P/CoP-2$, respectively. Charge/discharge curves of Co-based samples obtained under (**c**) different ratio of Zn to Co and (**d**) various temperature in 1 M KOH electrolyte at various current densities. The obtained Co_2P/CoP samples with the various temperature of 300°C and 500°C are denoted as CoP-300 and $Co_2P/CoP-500$, respectively

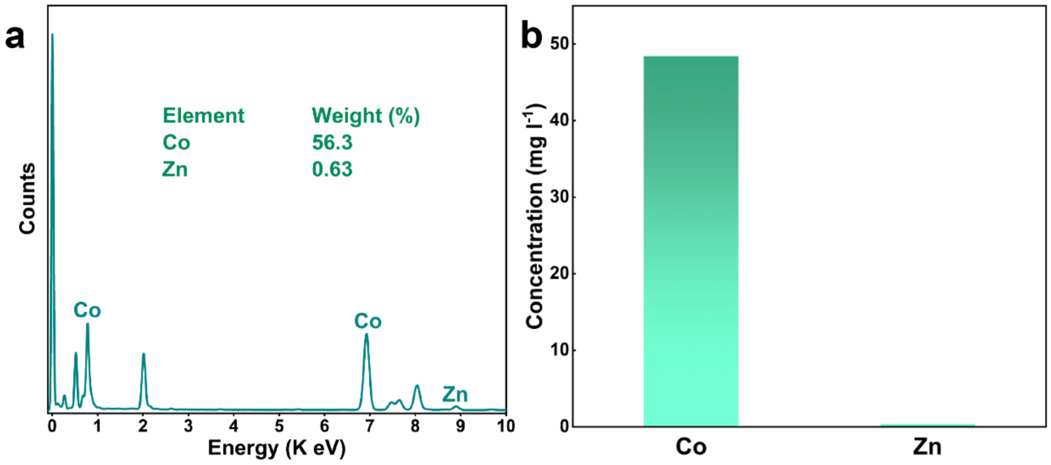

Fig. S11 (**a**) EDX analysis and (**b**) ICP-MS of the Co_2P/CoP NAs

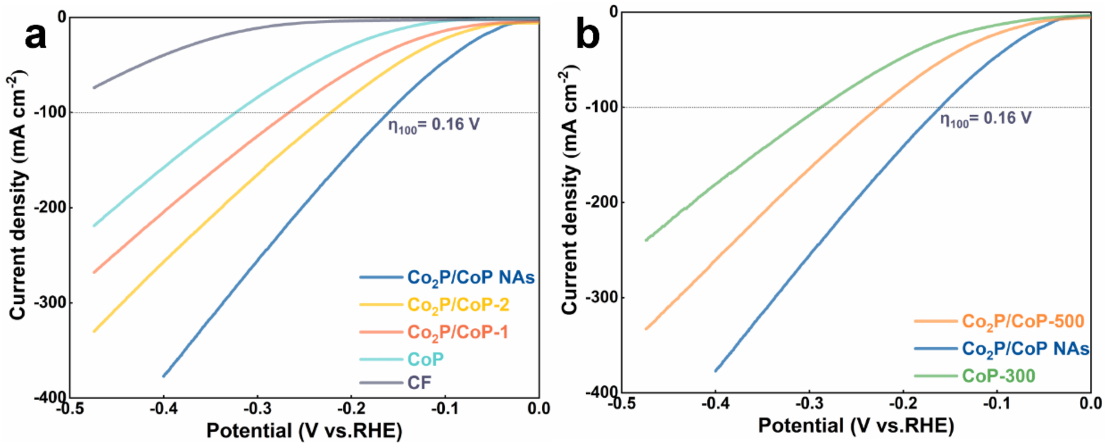

Fig. S12 Polarization curves of Co-based samples obtained under (**a**) different ratio of Zn to Co and (**b**) various temperature in 1 M KOH electrolyte. The obtained Co_2P/CoP samples with the molar ratio of Co to Zn (8 to 1 and 4 to 1) are denoted as Co_2P/CoP-1 and Co_2P/CoP-2, respectively. The obtained Co_2P/CoP samples with the various temperature of 300°C and 500 °C are denoted as CoP-300 and Co_2P/CoP-500, respectively

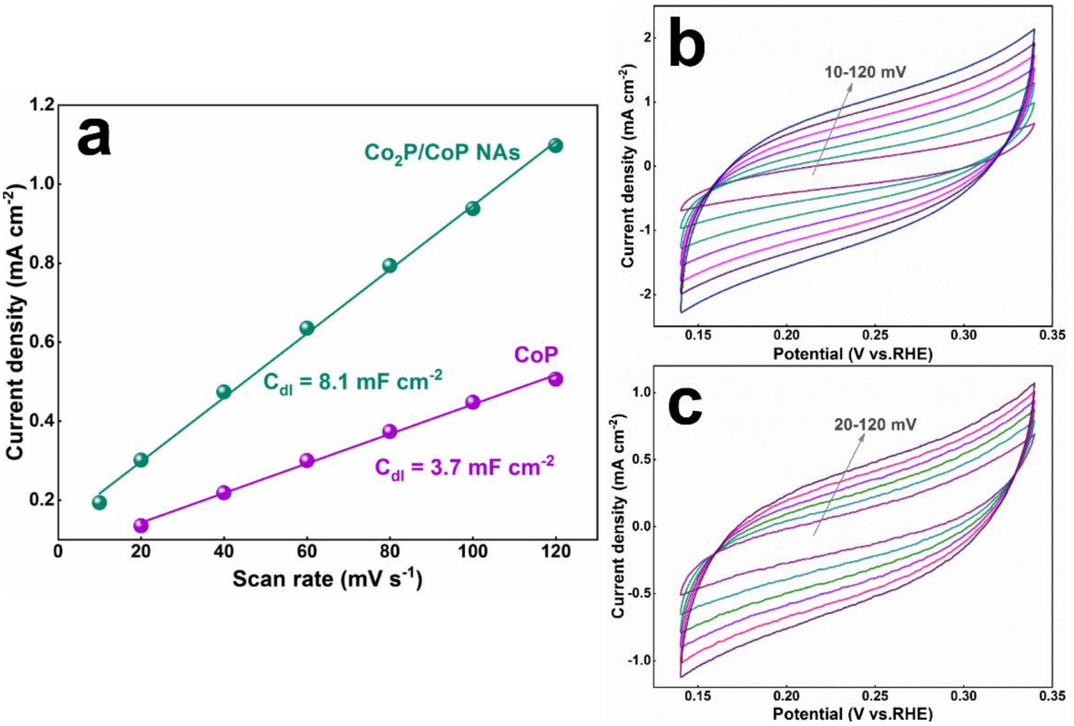

Fig. S13 (**a**) Capacitive current at 0.24 V *vs*. RHE as a function of scan rate for Co_2P/CoP NAs and CoP. Cyclic voltammograms (CV) for (**b**) Co_2P/CoP NAs and (**c**) CoP with different rates from 10 to 120 mV s^{-1} in the potential range of 0.14-0.34 V *vs*. RHE. The C_{dl} could be obtained according to the equation: $C_{dl} = I_c/\nu$, where C_{dl}, I_c, and ν are the double-layer capacitance (mF cm^{-2}) of the electroactive materials, charging current (mA cm^{-2}), and scan rate (mV s^{-1}), respectively.

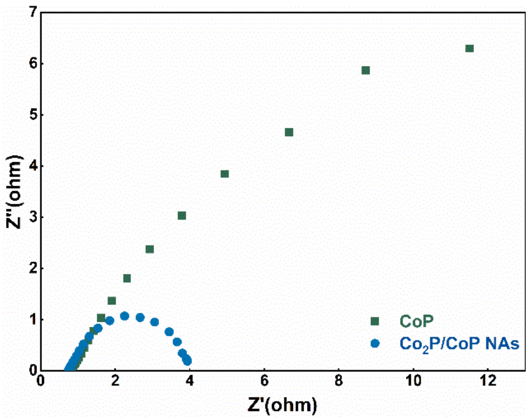

Fig. S14 EIS of the Co_2P/CoP NAs and CoP samples

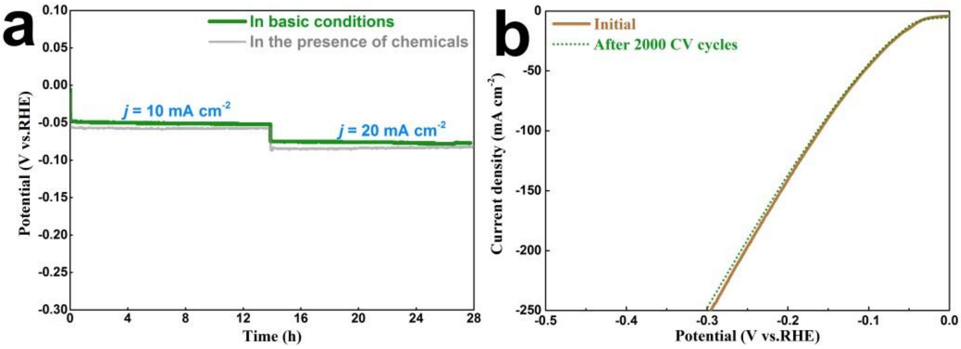

Fig. S15 (**a**) Time dependence of potential for the Co_2P/CoP NAs at the constant anodic current densities of 10 mA cm^{-2} and 20 mA cm^{-2} in two different electrolytes. (**b**) Stability measurement by recording the polarization curves for the Co_2P/CoP NAs after 2000 CV scans under basic condition

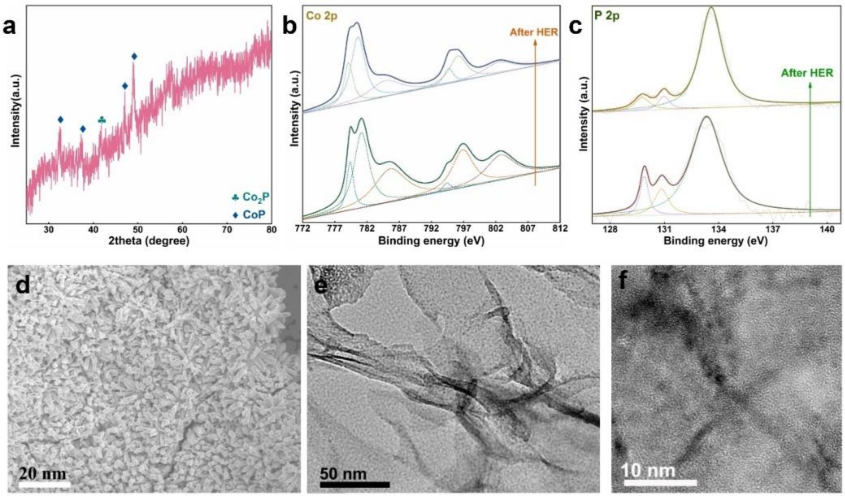

Fig. S16 Structural and morphological characterization of the Co_2P/CoP NAs after HER reaction. **a** XRD pattern of the Co_2P/CoP NAs after HER reaction. Comparison of High resolution XPS spectra of (**b**) Co and (**c**) P. (**d**) SEM image, (**e**) TEM image and HRTEM image of the Co_2P/CoP NAs after HER reaction

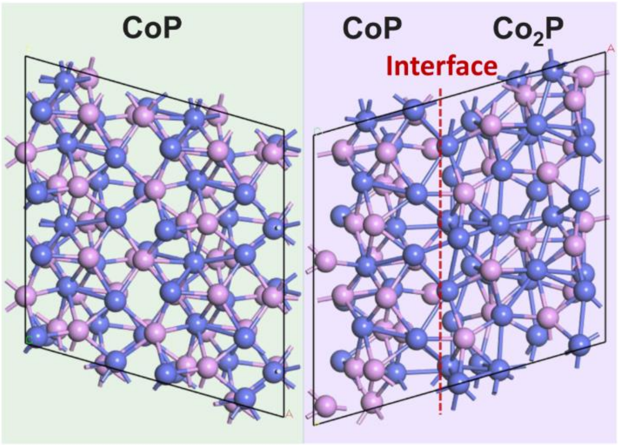

Fig. S17 Top view of the schematic models of pure CoP (111) and the optimized Co_2P(111)/CoP(111). Blue balls: Ni; pink balls: P

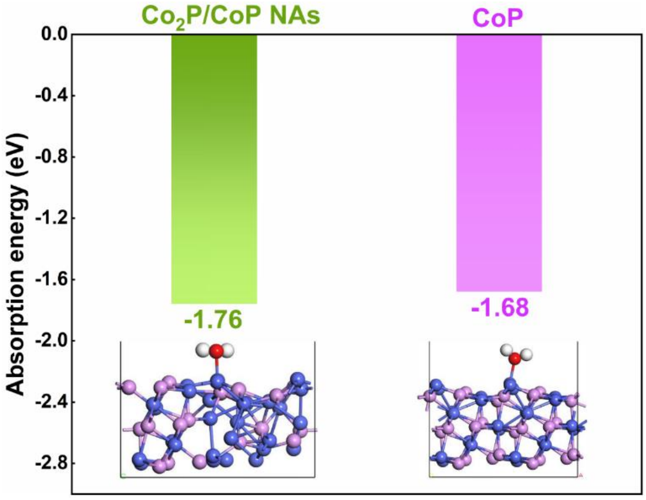

Fig. S18 Calculated absorption energy of H_2O on the surface of Co_2P/CoP and CoP

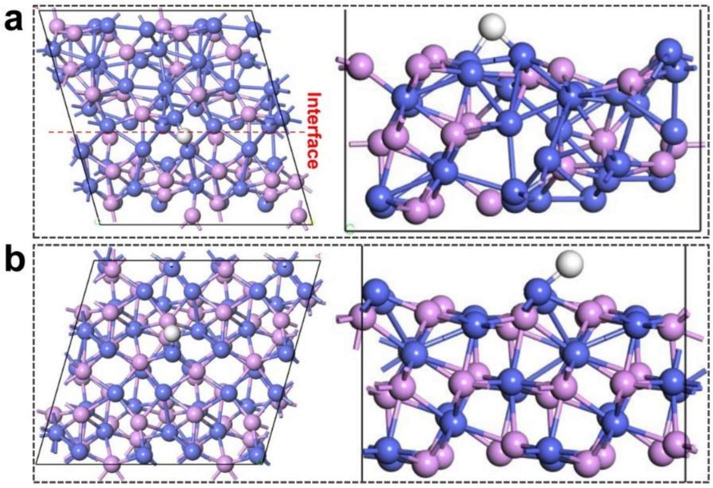

Fig. S19 Top (left) and side (right) view of the schematic models of (**a**) the optimized Co_2P(111)/CoP(111) and (**b**) pure CoP (111) with H* adsorbed on their surfaces

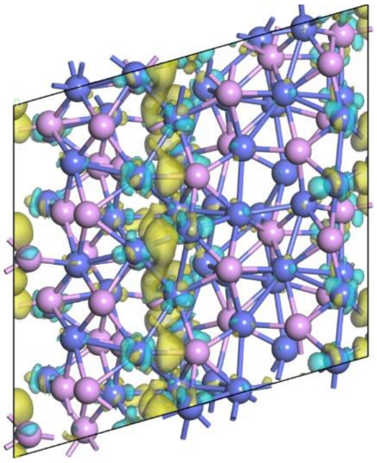

Fig. S20 Charge density difference plot at the $Co_2P(111)/CoP(111)$ interface; the yellow and light blue regions represent electron accumulation and depletion, respectively. Blue balls: Ni; pink balls: P

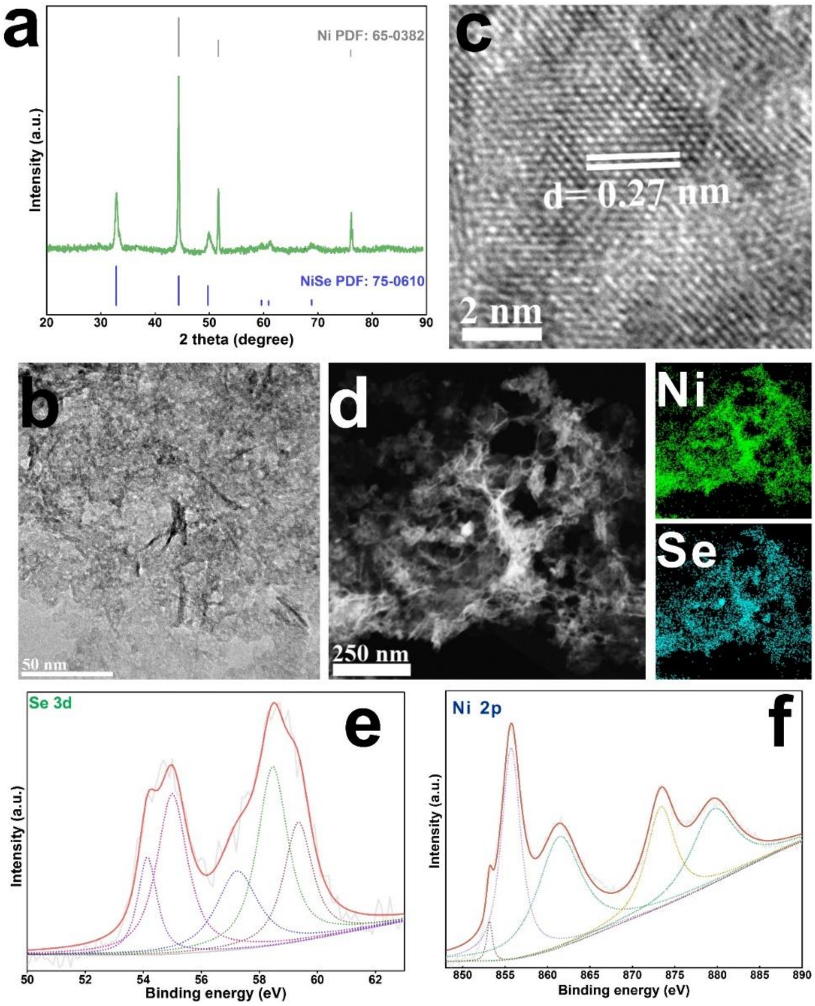

Fig. S21 (**a**) XRD pattern of NiSe. (**b-c**) TEM and HRTEM images with the element mapping of NiSe electrode (**d**). (**e-f**) High-resolution XPS spectrum of Se 3d and Ni 2p for the NiSe electrode

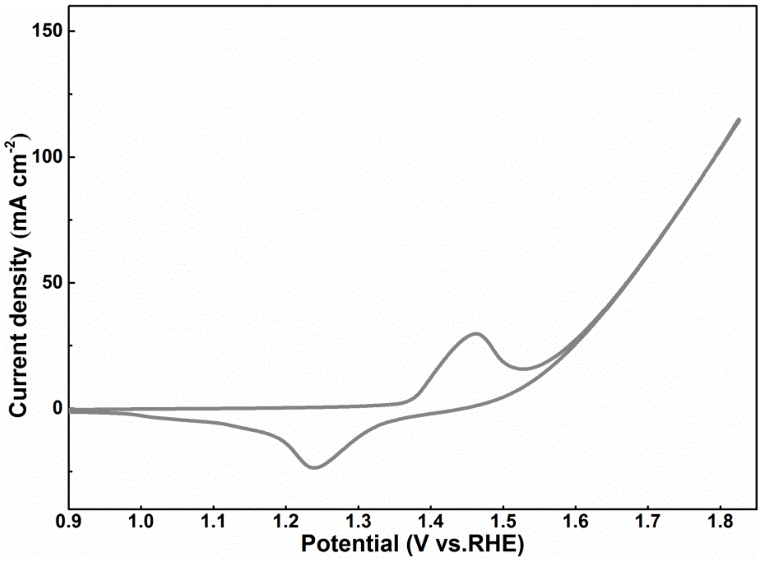

Fig. S22 CV curve of NiSe/NF under alkaline conditions

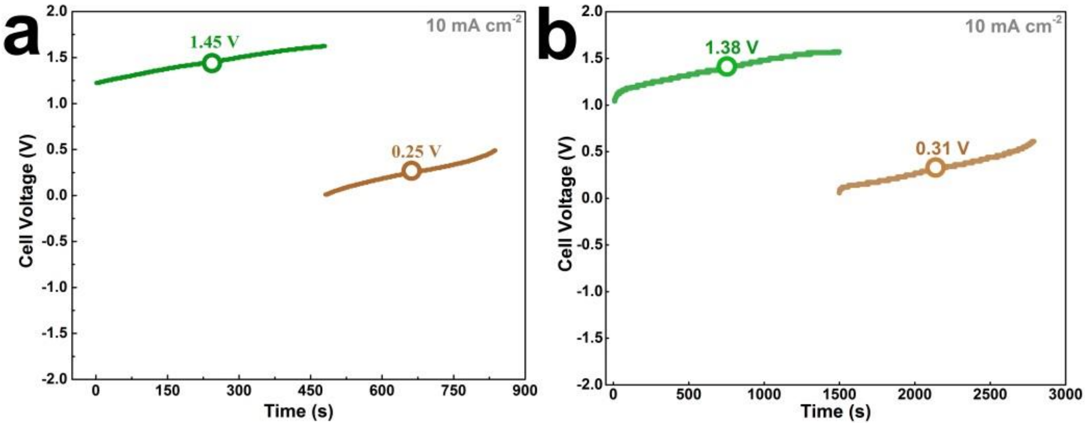

Fig. S23 Total driven voltages of (**a**) pure CoP and (**b**) Co_2P/CoP NAs electrode to support the current density of 10 mA cm^{-2} in 1.0 M KOH

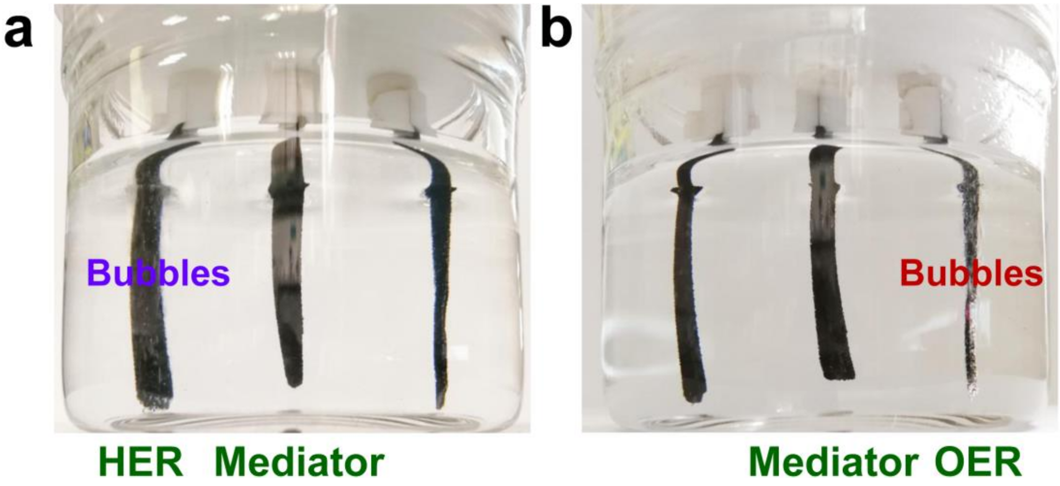

Fig. S24 Photo profiles of the H_2/O_2 generation in Steps 1(a) and 2 (b), where it is observed that H_2 and O_2 are produced on the HER (**a**) and OER (**b**) electrodes

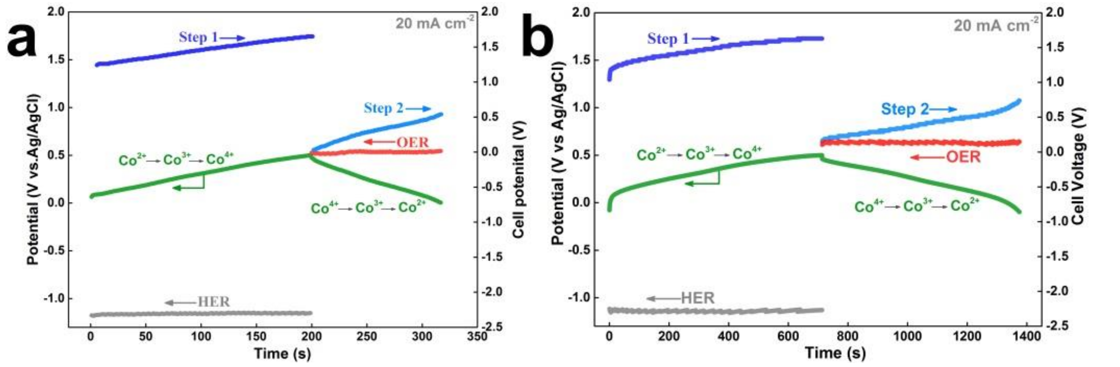

Fig. S25 Chronopotentiometry data (potential versus time) of batch-reactors using (**a**) pure CoP or (**b**) Co_2P/CoP NAs electrode. Chronopotentiometry curves were recorded at a current density of 20 mA cm^{-2}. [(Voltage of Step 1) = (Potential of Co charge) − (Potential of HER); (Voltage of Step 2) = (Potential of OER) − (Potential of Co discharge)]. Voltages for H_2 production (Step 1) and O_2 production (Step 2) are respectively labelled using the blue and indigo lines. Chronopotentiometry data (potential versus time) of HER electrode, mediator electrode, and OER electrode are labelled using the gray, green and red lines, respectively.

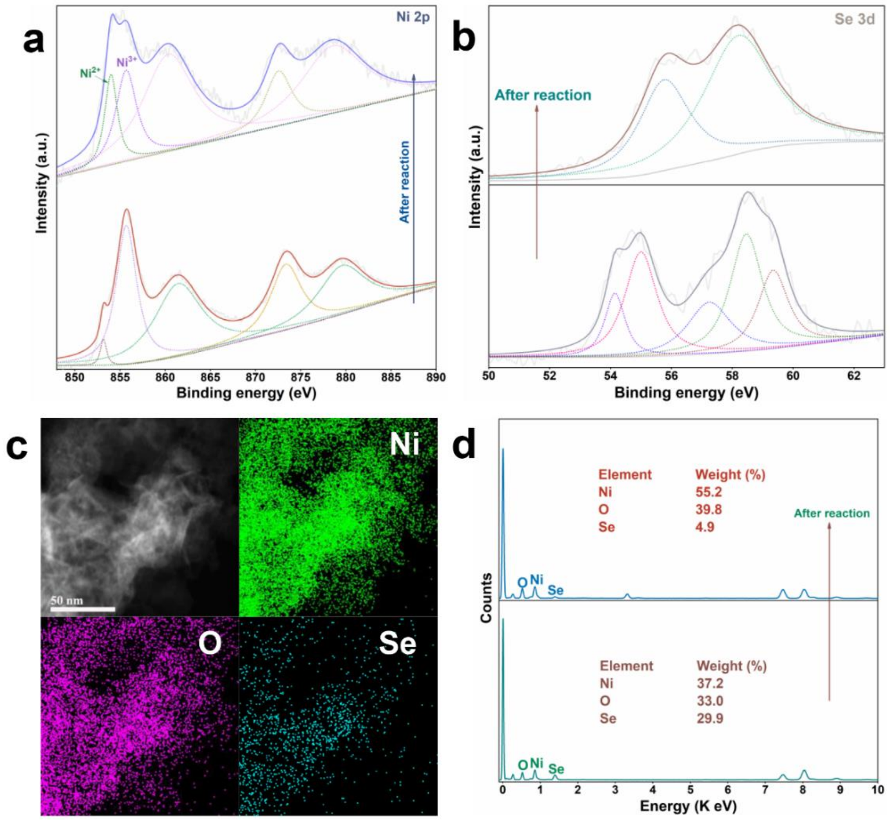

Fig. S26 Structural and morphological characterization of the NiSe after reaction. Comparison of High resolution XPS spectra of (**a**) Ni and (**b**) Se. (**c**) EDX mapping images of Ni, O, and Se and the corresponding EDX analysis of the NiSe after reaction

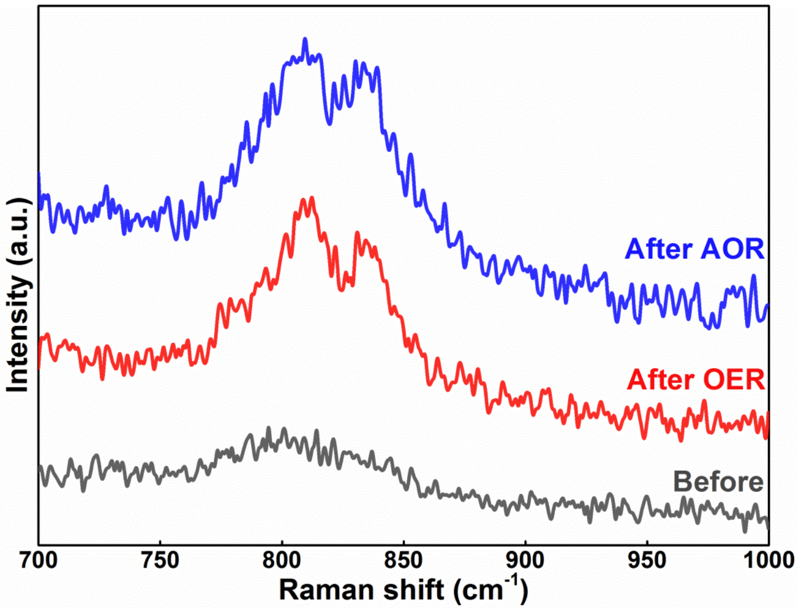

Fig. S27 Raman spectra of Co_2P/CoP NAs before and after OER or AOR electrolysis. AOR, ammonium oxidation reaction

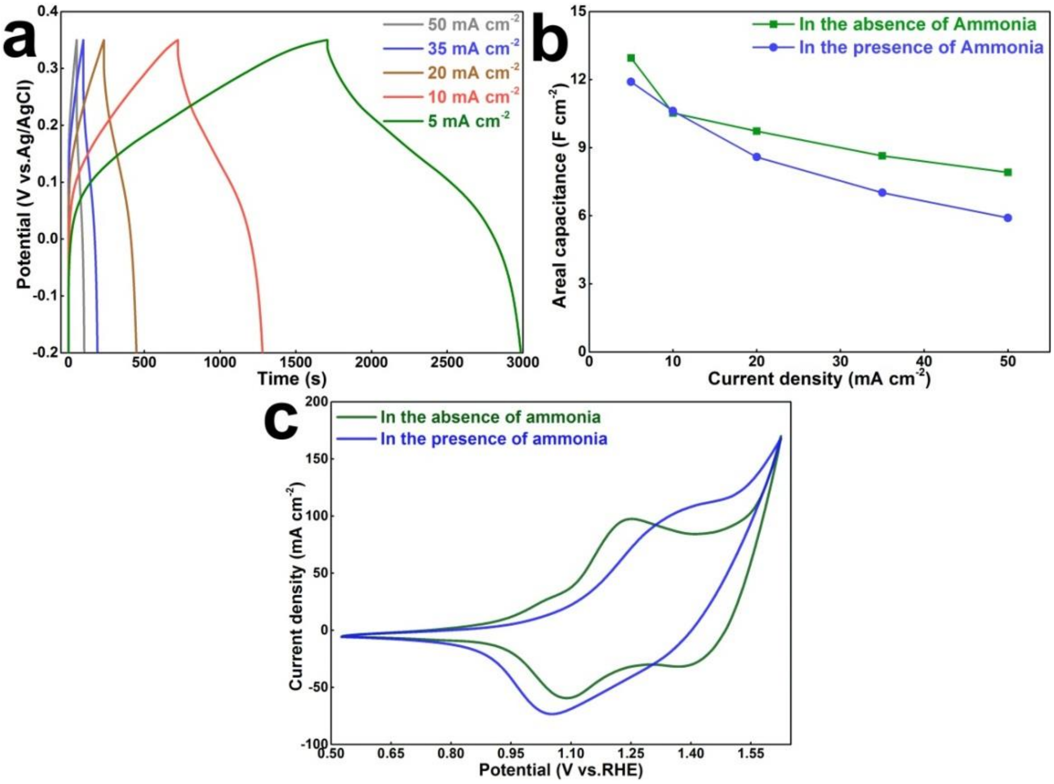

Fig. S28 (**a**) Galvanostatic charge/discharge curves for the Co_2P/CoP NAs electrode at different current densities in NH_3 containing solution. (**b**) Plots of areal capacitance versus current density of Co_2P/CoP NAs in two electrolytes. (**c**) CV curves of Co_2P/CoP NAs at a scan rate of 5 mV s^{-1} in two electrolytes

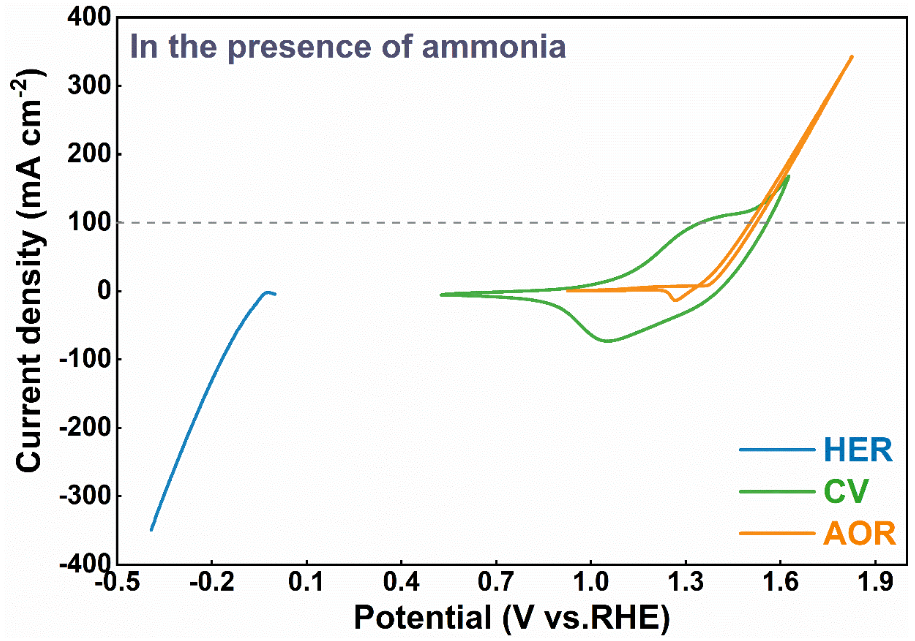

Fig. S29 Comparison of the curves of HER (blue line) and CV (green line) of Co_2P/CoP NAs as well as AOR (yellow line) of NiSe electrodes under alkaline-ammonia conditions

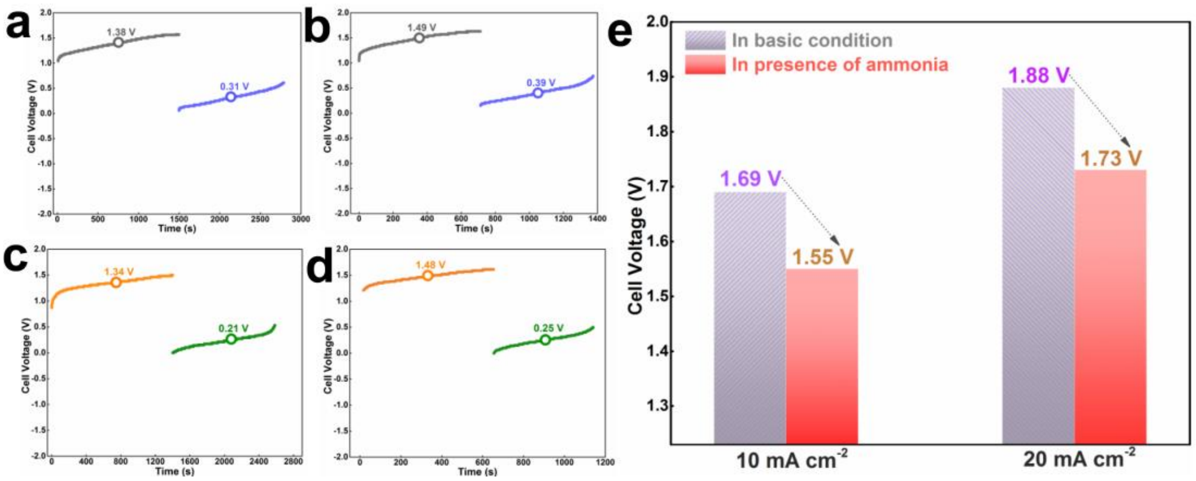

Fig. S30 Driving voltages with different step-time to support the current density of 10 mA cm^{-2} in (**a**)1.0 M KOH and (**c**) NH_3 containing solution. The driving voltages with different step-time to support the current density of 20 mA cm^{-2} in (**b**)1.0 M KOH and (**d**) NH_4^+ containing solution. (**e**) Comparison of total driving voltage (Step 1 + Step 2) to achieve benchmark current density in two electrolytes. The total driving voltages can be calculated via the average driving voltages of Step 1 + Step 2

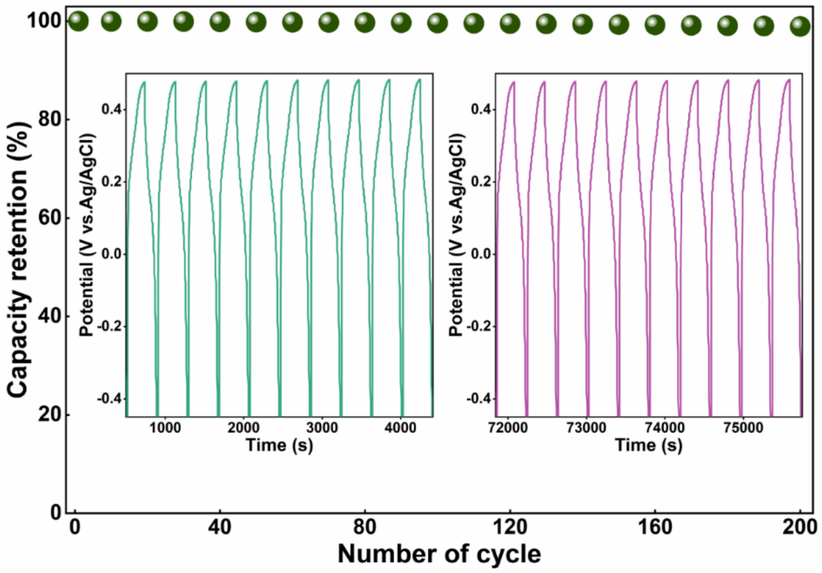

Fig. S31 Cyclic charge-discharge performance of Co_2P/CoP NAs at the current density of 50 mA; inset: corresponding first and last ten cycles showing retention of 98.9% charge storing ability even after 200 cycles

Supplementary Table

Table S1 Comparison of the HER performance for the Co_2P/CoP NAs catalyst with other reported electrocatalysts in alkaline electrolytes

HER Catalysts	Electrolyte	Tafel slope (mV dec^{-1})	Overpotential (mV) 10 mA cm^{-2}	Overpotential (mV) 100 mA cm^{-2}	Refs.
**Co_2P/CoP NAs**	**1 M KOH**	**57**	**40**	**160**	**This work**
**CoP/CoMoP**	1 M KOH	33	34	94	[S1]
**$CoSe_2$ NP**	1 M KOH	42.1	139	184	[S2]
**S:CoP**	1 M KOH	79	109	185	[S3]
**$Ni_{2(1-x)}Mo_{2x}P$**	1 M KOH	46.4	72	162	[S4]
**Cu NDs/Ni_3S_2 NTs**	1 M KOH	76.2	132	/	[S5]
**TiO_2@Ni_3S_2**	1 M KOH	69	112	177	[S6]
**$MoNi_4$/SSW**	1 M KOH	40	/	63	[S7]
**CoP/NiCoP**	1 M KOH	88	133	210	[S8]
**Cr-Co_4N NR**	1 M KOH	38.1	21	99	[S9]
**CMS/Ni**	1 M KOH	48.2	/	217	[S10]
**P-Fe_3O_4**	1 M KOH	41.9	42	138	[S11]
**Ni_3N-NV**	1 M KOH	37	64	218	[S12]
**N-Co_2P**	1 M KOH	51	34	/	[S13]

**B,N:Mo_2C@BCN-3**	1 M KOH	62	100	198	[S14]
**$Ni_2P/NiTe_2$**	1 M KOH	80	62	143	[S15]
**PW-Co_3N NWA**	1 M KOH	40	41	/	[S16]
**$CoP/Ni_5P_4/CoP$**	1 M KOH	58	71	140	[S17]
**N-$NiCo_2S_4$**	1 M KOH	41	37	/	[S18]
**MoO_2-FeP@C**	1 M KOH	48	103	196	[S19]

Table S2 Bader charge analysis and their differences to purely ionic models (ΔQ)

Sample	Atoms	Bader electrons	ΔQ
CoP(111)	Co_1	8.68	-0.32
	P_1	5.30	0.30
$Co_2P(111)/CoP(111)$	Co_1	8.60	-0.40
	Co_2	8.75	-0.25
	P_1	5.35	0.35
	P_2	5.41	0.41

Table S3 Comparison of our redox mediator for gas separation with reported gas separation systems with redox mediators

Redox mediator	Electrolyte	Separated gas	Current density (mA cm^{-2})	Average cell voltage (V)	Refs.
**Co_2P/CoP NAs**	**1 M KOH**	**H_2/O_2**	**10**	**1.69**	**This work**
			**20**	**1.88**	
	**1 M KOH + 1 M NH_3**	**H_2/N_2**	**10**	**1.55**	
			**20**	**1.73**	
**$Ni(OH)_2$**	1 M KOH	H_2/O_2	20	1.98	[S20]
			5	2.10	[S21]
**$Ni_{0.9}Co_{0.1}(OH)_2$**	5 M KOH	H_2/O_2	10	1.44	[S22]
			50	1.50	
**$Na_{0.44}MnO_2$**	1 M KOH (step 1) / Saturated NaCl (step 2)	H_2/Cl_2	10	2.37	[S23]
			20	2.51	
**PTPAn**	0.5 M H_2SO_4	H_2/O_2	1.66	1.65	[S24]
			33.3	2.22	
**PTO**	0.5 M H_2SO_4	H_2/O_2	1.66	1.66	[S25]
			16.6	1.87	

Supplementary References

[S1]X. Huang, X. Xu, X. Luan, D. Cheng, CoP nanowires coupled with CoMoP nanosheets as a highly efficient cooperative catalyst for hydrogen evolution reaction. Nano Energy **68**, 104332 (2020). https://doi.org/10.1016/j.nanoen.2019.104332

[S2]D. Kong, H. Wang, Z. Lu, Y. Cui, $CoSe_2$ nanoparticles grown on carbon fiber paper: an efficient and stable electrocatalyst for hydrogen evolution reaction. J. Am. Chem. Soc. **136**, 4897-4900 (2014). https://doi.org/10.1021/ja501497n

[S3]M. A. R. Anjum, M. S. Okyay, M. Kim, M. H. Lee, N. Park, J. S. Lee, Bifunctional sulfur-doped cobalt phosphide electrocatalyst outperforms all-noble-metal electrocatalysts in alkaline electrolyzer for overall water splitting. Nano Energy **53**, 286-295 (2018). https://doi.org/10.1016/j.nanoen.2018.08.064

[S4]L. Yu, I. K. Mishra, Y. Xie, H. Zhou, J. Sun, J. Zhou, Y. Ni, D. Luo, F. Yu, Y. Yu, S. Chen, Z. Ren, Ternary $Ni_{2(1-x)}Mo_{2x}P$ nanowire arrays toward efficient and stable hydrogen evolution electrocatalysis under large-current-density. Nano Energy **53**, 492-500 (2018). https://doi.org/10.1016/j.nanoen.2018.08.025

[S5]J. X. Feng, J. Q. Wu, Y. X. Tong, G. R. Li, Efficient hydrogen evolution on Cu nanodots-decorated Ni_3S_2 nanotubes by optimizing atomic hydrogen adsorption and desorption. J. Am. Chem. Soc. **140**, 610-617 (2018). https://doi.org/10.1021/jacs.7b08521

[S6]S. Deng, K. Zhang, D. Xie, Y. Zhang, Y. Zhang, Y. Wang, J. Wu, X. Wang, H. J. Fan, X. Xia, J. Tu, High-index-faceted Ni_3S_2 branch arrays as bifunctional electrocatalysts for efficient water splitting. Nano-Micro Lett. **11**, 12 (2019). https://doi.org/10.1007/s40820-019-0242-8

[S7]V. R. Jothi, K. Karuppasamy, T. Maiyalagan, H. Rajan, C. Y. Jung, S. C. Yi, Corrosion and alloy engineering in rational design of high current density electrodes for efficient water splitting. Adv. Energy Mater. **10**, 1904020 (2020). https://doi.org/10.1002/aenm.201904020

[S8]Y. Lin, K. Sun, S. Liu, X. Chen, Y. Cheng, W. C. Cheong, Z. Chen, L. Zheng, J. Zhang, X. Li, Y. Pan, C. Chen, Construction of CoP/NiCoP nanotadpoles heterojunction interface for wide pH hydrogen evolution electrocatalysis and supercapacitor. Adv. Energy Mater. **9**, 1901213 (2019). https://doi.org/10.1002/aenm.201901213

[S9]N. Yao, P. Li, Z. Zhou, Y. Zhao, G. Cheng, S. Chen, W. Luo, Synergistically tuning water and hydrogen binding abilities over Co_4N by Cr doping for exceptional alkaline hydrogen evolution electrocatalysis. Adv. Energy Mater. **9**, 1902449 (2019). https://doi.org/10.1002/aenm.201902449

[S10] J. Li, W. Xu, J. Luo, D. Zhou, D. Zhang, L. Wei, P. Xu, D. Yuan, Synthesis of 3D hexagram-like cobalt-manganese sulfides nanosheets grown on nickel foam: A bifunctional electrocatalyst for overall water splitting. Nano-Micro Lett. **10**, 6 (2018). https://doi.org/10.1007/s40820-017-0160-6

[S11] J. Zhang, X. Shang, H. Ren, J. Chi, H. Fu, B. Dong, C. Liu, Y. Chai, Modulation of inverse spinel Fe_3O_4 by phosphorus doping as an industriallypromising electrocatalyst for hydrogen evolution. Adv. Mater. **31**, 1905107 (2019). https://doi.org/10.1002/adma.201905107

[S12] H. Yan, Y. Xie, A. Wu, Z. Cai, L. Wang, C. Tian, X. Zhang, H. Fu, Anion-modulated HER and OER activities of 3D Ni-V-based interstitial compound heterojunctions for high-efficiency and stable overall water splitting. Adv. Mater. **31**, 1901174 (2019). https://doi.org/10.1002/adma.201901174

[S13] Y. Men, P. Li, J. Zhou, G. Cheng, S. Chen, W. Luo, Tailoring the electronic structure of Co_2P by N doping for boosting hydrogen evolution reaction at all pH values. ACS Catal. **9**, 3744-3752 (2019). https://doi.org/10.1021/acscatal.9b00407

[S14] M. A. R. Anjum, M. H. Lee, J. S. Lee, Boron- and nitrogen-codoped molybdenum carbide nanoparticles imbedded in a BCN network as a bifunctional electrocatalyst for hydrogen and oxygen evolution reactions. ACS Catal. **8**, 8296-8305 (2018). https://doi.org/10.1021/acscatal.8b01794

[S15] Y. Li, X. Tan, H. Tan, H. Ren, S. Chen, W. Yang, S. C. Smith, C. Zhao, Phosphine vapor-assisted construction of heterostructured $Ni_2P/NiTe_2$ catalysts for efficient hydrogen evolution. Energy Environ. Sci. (2020) DOI: 10.1039/d0ee00666a. https://doi.org/10.1039/D0EE00666A

[S16] Y. Liu, J. Zhang, Y. Li, Q. Qian, Z. Li, Y. Zhu, G. Zhang, Manipulating dehydrogenation kinetics through dual-doping Co_3N electrode enables highly efficient hydrazine oxidation assisting self-powered H_2 production. Nat. Commun. **11**, 1853 (2020). https://doi.org/10.1038/s41467-020-15563-8

[S17] I. K. Mishra, H. Zhou, J. Sun, F. Qin, K. Dahal, J. Bao, S. Chen, Z. Ren, Hierarchical $CoP/Ni_5P_4/CoP$ microsheet arrays as a robust pH-universal electrocatalyst for efficient hydrogen generation. Energy Environ. Sci. **11**, 2246-2252 (2018). https://doi.org/10.1039/C8EE01270A

[S18] Y. Wu, X. Liu, D. Han, X. Song, L. Shi, Y. Song, S. Niu, Y. Xie, J. Cai, S. Wu, J. Kang, J. Zhou, Z. Chen, X. Zheng, X. Xiao, G. Wang, Electron density modulation of $NiCo_2S_4$ nanowires by nitrogen incorporation for highly efficient hydrogen evolution catalysis. Nat. Commun. **9**, 1425 (2018). https://doi.org/10.1038/s41467-018-03858-w

[S19] G. Yang, Y. Jiao, H. Yan, Y. Xie, A. Wu, X. Dong, D. Guo, C. Tian, H. Fu, Interfacial engineering of MoO_2-FeP heterojunction for highly efficient hydrogen evolution coupled with biomass electrooxidation. Adv. Mater. **32**, 2000455 (2020). https://doi.org/10.1002/adma.202000455

[S20] L. Chen, X. Dong, Y. Wang, Y. Xia, Separating hydrogen and oxygen evolution in alkaline water electrolysis using nickel hydroxide. Nat. Commun. **7**, 11741 (2016). https://doi.org/10.1038/ncomms11741

[S21] A. Landman, H. Dotan, G. E. Shter, M. Wullenkord, A. Houaijia, A. Maljusch, G. S. Grader, A. Rothschild, Photoelectrochemical water splitting in separate oxygen and hydrogen cells. Nat. Mater. **16**, 646-651 (2017). https://doi.org/10.1038/nmat4876

[S22] H. Dotan, A. Landman, S. W. Sheehan, K. D. Malviya, G. E. Shter, D. A. Grave, Z. Arzi, N. Yehudai, M. Halabi, N. Gal, N. Hadari, C. Cohen, A. Rothschild, G. S. Grader, Decoupled hydrogen and oxygen evolution by a two-step electrochemical-chemical cycle for efficient overall water splitting. Nat. Energy **4**, 786-795 (2019). https://doi.org/10.1038/s41560-019-0462-7

[S23] M. Hou, L. Chen, Z. Guo, X. Dong, Y. Wang, Y. Xia, A clean and membrane-free chlor-alkali process with decoupled Cl_2 and H_2/NaOH production. Nat. Commun. **9**, 438 (2018). https://doi.org/10.1038/s41467-018-02877-x

[S24] Y. Ma, X. Dong, Y. Wang, Y. Xia, Decoupling hydrogen and oxygen production in acidic water electrolysis using a polytriphenylamine-based battery electrode. Angew. Chem. Int. Ed. **57**, 2904-2908 (2018). https://doi.org/10.1002/anie.201800436

[S25] Y. Ma, Z. Guo, X. Dong, Y. Wang, Y. Xia, Organic proton-buffer electrode to separate hydrogen and oxygen evolution in acid water electrolysis. Angew. Chem. Int. Ed. **58**, 4622-4626 (2019). https://doi.org/10.1002/anie.201814625
